# Supplementary material for: The potential shared role of inflammation in insulin resistance and schizophrenia: A bidirectional two-sample mendelian randomization study
Source: PLoS Med. 2021 Mar 12;18(3):e1003455. doi: 10.1371/journal.pmed.1003455 (PMC7954314; doi:10.1371/journal.pmed.1003455)
Supplement: S1 Methods — (DOCX) [file pmed.1003455.s001.docx]

**The potential shared role of inflammation in insulin resistance and schizophrenia: A bi-directional two-sample Mendelian randomization study**

Perry B.I. *et al*

**S1 Methods: Directed Acyclic Graphs Outlining Potential Mechanisms of Association between Inflammation, Insulin Resistance and Schizophrenia**

Inflammation

Insulin Resistance

Schizophrenia

Inflammation

Insulin Resistance

Schizophrenia

Inflammation

Insulin Resistance

Schizophrenia

Inflammation

Insulin Resistance

Schizophrenia

A

B

C

D

A=Represents a mechanism whereby inflammation is a common cause (confounder) for insulin resistance and schizophrenia; B=Represents a mechanism whereby insulin resistance mediates an association between inflammation and schizophrenia; C=Represents a mechanism whereby inflammation is a common cause (confounder) for schizophrenia and insulin resistance; D=Represents a mechanism whereby schizophrenia mediates an association between inflammation and insulin resistance.
