# Supplementary material for: The potential shared role of inflammation in insulin resistance and schizophrenia: A bidirectional two-sample mendelian randomization study
Source: PLoS Med. 2021 Mar 12;18(3):e1003455. doi: 10.1371/journal.pmed.1003455 (PMC7954314; doi:10.1371/journal.pmed.1003455)
Supplement: S2 Methods — (DOCX) [file pmed.1003455.s002.docx]

**The potential shared role of inflammation in insulin resistance and schizophrenia: A bi-directional two-sample Mendelian randomization study**

Perry B.I. *et al*

**S2 Methods: GWAS used for SNP Selection**

| **Cardiometabolic Exposure** | **Author (Year)** | **Consortia** | **Ethnic Origin** | **Sample** | **Setting^a^** | **GWAS-significant SNPs, No.** | **Inflammation-related SNPs, No.^b^** |
| --- | --- | --- | --- | --- | --- | --- | --- |
| Fasting Insulin [1] | Lotta et al (2016) | MAGIC | European | 108,557 (101,393 adults and 7,164 adolescents) | Meta-GWAS of 19 European Cohort Studies, participants with diabetes excluded | 53 | 5 |
| Triglycerides [1] | Lotta et al (2016) | EPIC-InterAct, FPLD1 | European | 188,577 adults | Meta-GWAS of 45 population-based cohort studies and case-control studies. | 53 | 5 |
| HDL [1] | Lotta et al (2016) | EPIC-InterAct, FPLD1 | European | 188,577 adults | Meta-GWAS of 45 population-based cohort studies and case-control studies. | 53 | 4 |
| LDL [2] | Willer et al (2013) | GLGC | European, East Asian, South Asian, African | 173,082 adults | Meta-GWAS of 45 population-based cohort studies and case-control studies. | 79 | 13 |
| Fasting Plasma Glucose [3] | Manning et al (2012) | MAGIC | European | 58,074 adults | Meta-GWAS of 29 European population-based cohort studies. participants with diabetes excluded. | 22 | 2 |
| T2DM [4] | Mahajan et al (2018) | DIAGRAM | European, East Asian, South Asian, Mexican, Mexican American | 435,387 adults;  (81,412 with T2DM and 370,832 controls) | Meta-GWAS of two large prospective European cohort studies | 152 | 7 |
| BMI [5] | Locke et al (2015) | GIANT | European, African, Asian | 339,224 adults | Meta-GWAS of 125 European cohort studies, adjusted for age | 97 | 6 |
| HbA1C [6] | Wheeler et al (2017) | MAGIC | European, African American, East Asian, South Asian | 159,940 adults | Meta-GWAS of 82 population-based cohort studies. Participants with diabetes excluded. | 60 | 7 |
| Glucose Tolerance [7] | Saxena et al (2010) | MAGIC | European | 15,234 adults | Meta-GWAS of 9 population-based cohort studies. Participants with diabetes excluded. | 7 | 0 |
| Leptin [8] | Kilpelainen et al (2016) | - | European | 82,315 adults | Meta-GWAS of 32 population-based cohort studies adjusted for age and sex. | 5 | 0 |

SNP=Single Nucleotide Polymorphism; GWAS=Genome-Wide Association Study; HDL=High-Density Lipoprotein; LDL=Low-Density Lipoprotein; T2DM=Type 2 Diabetes Mellitus; BMI=Body Mass Index; HbA1C=Glycated Haemoglobin; MAGIC=Meta-Analysis of Glucose and Insulin Related traits Consortium; GLGC=Global Lipids Genetics Consortium; DIAGRAM=Diabetes Genetics Replication and Meta-Analysis; GIANT=Genetic Investigation of Anthropometric Traits
^a^See original GWAS publication for detailed demographic and setting information for studies included in meta-GWAS.
^b^Number of SNPs with pleiotropy for inflammation at genome-wide significance

**Reference**

1. Lotta LA, Gulati P, Day FR, Payne F, Ongen H, van de Bunt M, et al. Integrative genomic analysis implicates limited peripheral adipose storage capacity in the pathogenesis of human insulin resistance. Nat Genet. 2017;49(1):17-26.

2. Willer CJ, Schmidt EM, Sengupta S, Peloso GM, Gustafsson S, Kanoni S, et al. Discovery and refinement of loci associated with lipid levels. Nat Genet. 2013;45(11):1274-83.

3. Manning AK, Hivert MF, Scott RA, Grimsby JL, Bouatia-Naji N, Chen H, et al. A genome-wide approach accounting for body mass index identifies genetic variants influencing fasting glycemic traits and insulin resistance. Nat Genet. 2012;44(6):659-69.

4. Mahajan A, Wessel J, Willems SM, Zhao W, Robertson NR, Chu AY, et al. Refining the accuracy of validated target identification through coding variant fine-mapping in type 2 diabetes. Nat Genet. 2018;50(4):559-71.

5. Locke AE, Kahali B, Berndt SI, Justice AE, Pers TH, Day FR, et al. Genetic studies of body mass index yield new insights for obesity biology. Nature. 2015;518(7538):197-206.

6. Wheeler E, Leong A, Liu CT, Hivert MF, Strawbridge RJ, Podmore C, et al. Impact of common genetic determinants of Hemoglobin A1c on type 2 diabetes risk and diagnosis in ancestrally diverse populations: A transethnic genome-wide meta-analysis. PLoS Med. 2017;14(9):e1002383.

7. Saxena R, Hivert MF, Langenberg C, Tanaka T, Pankow JS, Vollenweider P, et al. Genetic variation in GIPR influences the glucose and insulin responses to an oral glucose challenge. Nat Genet. 2010;42(2):142-8.

8. Kilpeläinen TO, Carli JF, Skowronski AA, Sun Q, Kriebel J, Feitosa MF, et al. Genome-wide meta-analysis uncovers novel loci influencing circulating leptin levels. Nat Commun. 2016;7:10494.
