# Supplementary material for: The potential shared role of inflammation in insulin resistance and schizophrenia: A bidirectional two-sample mendelian randomization study
Source: PLoS Med. 2021 Mar 12;18(3):e1003455. doi: 10.1371/journal.pmed.1003455 (PMC7954314; doi:10.1371/journal.pmed.1003455)
Supplement: S3 Methods — (DOCX) [file pmed.1003455.s003.docx]

**The potential shared role of inflammation in insulin resistance and schizophrenia: A bi-directional two-sample Mendelian randomization study**

Perry B.I. *et al*

**S3 Methods: SNPs used as instruments for fasting insulin, triglycerides and high-density lipoprotein**

| rs1011685 |
| --- |
| rs10195252 |
| rs2126259 |
| rs2745353 |
| rs2943645 |
| rs3822072 |
| rs3861397 |
| rs459193 |
| rs4804311 |
| rs7973683 |
| rs4976033 |
| rs683135 |
| rs731839 |
| rs972283 |

**Fasting Insulin Triglycerides HDL**

| rs10195252 |
| --- |
| rs2126259 |
| rs2943645 |
| rs308971 |
| rs3822072 |
| rs459193 |
| rs4846565 |
| rs4865796 |
| rs731839 |

| rs1011685 |
| --- |
| rs10195252 |
| rs132985 |
| rs2699429 |
| rs3861397 |
| rs7973683 |
| rs731839 |
| rs4804311 |
| rs2943645 |
