# Supplementary material for: The potential shared role of inflammation in insulin resistance and schizophrenia: A bidirectional two-sample mendelian randomization study
Source: PLoS Med. 2021 Mar 12;18(3):e1003455. doi: 10.1371/journal.pmed.1003455 (PMC7954314; doi:10.1371/journal.pmed.1003455)
Supplement: S4 Methods — (DOCX) [file pmed.1003455.s004.docx]

**The potential shared role of inflammation in insulin resistance and schizophrenia: A bi-directional two-sample Mendelian randomization study**

Perry B.I. *et al*

**S4 Methods: SNPs used as instruments for fasting plasma glucose**

| \| rs10276674 \| \| --- \| \| rs10830963 \| \| rs10974438 \| \| rs11039138 \| \| rs11195502 \| | \| rs11603334 \| \| --- \| \| rs11605924 \| \| rs11708067 \| \| rs17747324 \|   rs11558471 | \| rs2191349 \| \| --- \| \| rs2524299 \| \| rs2908282 \| \| rs4148804 \| | \| rs4869272 \| \| --- \| \| rs560887 \| \| rs6113722 \| \| rs7173964 \| | \| rs7644261 \| \| --- \| \| rs780093 \| \| rs882020 \| \| rs983309 \| |
| --- | --- | --- | --- | --- | --- | --- | --- | --- | --- | --- | --- | --- | --- | --- | --- | --- | --- | --- | --- | --- | --- | --- | --- | --- | --- |
