# Supplementary material for: The potential shared role of inflammation in insulin resistance and schizophrenia: A bidirectional two-sample mendelian randomization study
Source: PLoS Med. 2021 Mar 12;18(3):e1003455. doi: 10.1371/journal.pmed.1003455 (PMC7954314; doi:10.1371/journal.pmed.1003455)
Supplement: S5 Methods — (DOCX) [file pmed.1003455.s005.docx]

**The potential shared role of inflammation in insulin resistance and schizophrenia: A bi-directional two-sample Mendelian randomization study**

Perry B.I. *et al*

**S5 Methods: SNPs used as instruments for type 2 diabetes mellitus**

| rs1060105  rs1127787  rs1169288  rs1260326  rs13266634  rs140386498  rs1800437 | rs1801212  rs1801282  rs2032844  rs2073721  rs2276853  rs2296172  rs1800961 | rs328  rs35169799  rs35658696  rs35720761  rs3764002  rs5219  rs56200889 | rs60980157  rs665268  rs6762208  rs72928978  rs738409  rs7572857  rs58542926 | rs7607980  rs781831  rs9379084  rs9891146  rs2307111  rs28265 |
| --- | --- | --- | --- | --- |
