# Supplementary material for: The potential shared role of inflammation in insulin resistance and schizophrenia: A bidirectional two-sample mendelian randomization study
Source: PLoS Med. 2021 Mar 12;18(3):e1003455. doi: 10.1371/journal.pmed.1003455 (PMC7954314; doi:10.1371/journal.pmed.1003455)
Supplement: S6 Methods — (DOCX) [file pmed.1003455.s006.docx]

**The potential shared role of inflammation in insulin resistance and schizophrenia: A bi-directional two-sample Mendelian randomization study**

Perry B.I. *et al*

**S6 Methods: SNPs used as instruments for body mass index**

| rs1000940  rs10132280  rs1016287  rs10182181  rs10733682  rs10840100  rs11030104  rs11165643  rs11663558  rs11672660  rs1167827  rs11727676  rs12286929  rs12429545  rs12448257  rs12940622  rs12986742  rs13021737  rs13078960 | rs13107325  rs13130484  rs13191362  rs13201877  rs13329567  rs1421085  rs1441264  rs1460676  rs14810  rs1516725  rs1528435  rs16851483  rs17001654  rs17066856  rs17094222  rs17203016  rs17381664  rs17724992  rs1928295 | rs205262  rs2060604  rs2112347  rs2176040  rs2176598  rs2183825  rs2245368  rs2365389  rs2820292  rs2890652  rs3736485  rs3817334  rs3849570  rs3888190  rs4740619  rs4889606  rs492400  rs4981693  rs543874 | rs6091540  rs6465468  rs6477694  rs6567160  rs657452  rs6804842  rs7138803  rs7144011  rs7239883  rs7531118  rs7550711  rs7599312  rs7715256  rs7899106  rs7903146  rs879620  rs9304665  rs9374842  rs9400239 | rs9540493  rs9579083  rs977747  rs9926784  rs2033732  rs943005 |
| --- | --- | --- | --- | --- |
