# Supplementary material for: The potential shared role of inflammation in insulin resistance and schizophrenia: A bidirectional two-sample mendelian randomization study
Source: PLoS Med. 2021 Mar 12;18(3):e1003455. doi: 10.1371/journal.pmed.1003455 (PMC7954314; doi:10.1371/journal.pmed.1003455)
Supplement: S7 Methods — (DOCX) [file pmed.1003455.s007.docx]

**The potential shared role of inflammation in insulin resistance and schizophrenia: A bi-directional two-sample Mendelian randomization study**

Perry B.I. *et al*

**S7 Methods: SNPs used as instruments for glucose tolerance**

| \| rs1019503 \| \| --- \| \| rs11672660 \| | \| rs11717195 \| \| --- \| \| rs11782386 \| | \| rs12255372 \| \| --- \| \| rs6547829 \| | \| rs6975024 \| \| --- \| |
| --- | --- | --- | --- | --- | --- | --- | --- | --- | --- | --- |
