# Supplementary material for: The potential shared role of inflammation in insulin resistance and schizophrenia: A bidirectional two-sample mendelian randomization study
Source: PLoS Med. 2021 Mar 12;18(3):e1003455. doi: 10.1371/journal.pmed.1003455 (PMC7954314; doi:10.1371/journal.pmed.1003455)
Supplement: S8 Methods — (DOCX) [file pmed.1003455.s008.docx]

**The potential shared role of inflammation in insulin resistance and schizophrenia: A bi-directional two-sample Mendelian randomization study**

Perry B.I. *et al*

**S8 Methods: SNPs used as instruments for low density lipoprotein**

| \| rs10195252 \| \| --- \| \| rs10490626 \| \| rs10832962 \| \| rs10893499 \| \| rs10903129 \| \| rs112201728 \| \| rs11563251 \| \| rs11591147 \| \| rs1169288 \| \| rs12066643 \| \| rs1250229 \| \| rs12721109 \| \| rs12748152 \| \| rs12916 \| \| rs13206249 \|   rs6709904 | \| rs13277801 \| \| --- \| \| rs1367117 \| \| rs1408272 \| \| rs1564348 \| \| rs16831243 \| \| rs16891156 \| \| rs17404153 \| \| rs174583 \| \| rs1800961 \| \| rs1801689 \| \| rs1883025 \| \| rs2000999 \| \| rs2030746 \| \| rs2073547 \| \| rs2228603 \| \| rs2315065 \| | \| rs2328223 \| \| --- \| \| rs2390536 \| \| rs2419604 \| \| rs247616 \| \| rs2495495 \| \| rs2587534 \| \| rs2642438 \| \| rs267733 \| \| rs2710642 \| \| rs2737252 \| \| rs2738459 \| \| rs2886232 \| \| rs2954029 \| \| rs2965157 \| \| rs314253 \| \| rs3184504 \| | \| rs364585 \| \| --- \| \| rs3757354 \| \| rs3780181 \| \| rs4253776 \| \| rs4530754 \| \| rs4722551 \| \| rs4942486 \| \| rs4970712 \| \| rs5763662 \| \| rs579459 \| \| rs6016373 \| \| rs6065311 \| \| rs646776 \| \| rs6504872 \| \| rs6511720 \| \| rs6544713 \| \|  \| | \| \| rs676388 \| \| --- \| \| rs6818397 \| \| rs6882076 \| \| rs6909746 \| \| rs7254892 \| \| rs72902576 \| \| rs7534572 \| \| rs7551981 \| \| rs75687619 \| \| rs7640978 \| \| rs7832643 \| \| rs8017377 \| \| rs964184 \| \| rs9875338 \| \| rs9987289 \| \| \| --- \| --- \| --- \| --- \| --- \| --- \| --- \| --- \| --- \| --- \| --- \| --- \| --- \| --- \| --- \| --- \| |
| --- | --- | --- | --- | --- | --- | --- | --- | --- | --- | --- | --- | --- | --- | --- | --- | --- | --- | --- | --- | --- | --- | --- | --- | --- | --- | --- | --- | --- | --- | --- | --- | --- | --- | --- | --- | --- | --- | --- | --- | --- | --- | --- | --- | --- | --- | --- | --- | --- | --- | --- | --- | --- | --- | --- | --- | --- | --- | --- | --- | --- | --- | --- | --- | --- | --- | --- | --- | --- | --- | --- | --- | --- | --- | --- | --- | --- | --- | --- | --- | --- | --- | --- | --- | --- |
