# Supplementary material for: The potential shared role of inflammation in insulin resistance and schizophrenia: A bidirectional two-sample mendelian randomization study
Source: PLoS Med. 2021 Mar 12;18(3):e1003455. doi: 10.1371/journal.pmed.1003455 (PMC7954314; doi:10.1371/journal.pmed.1003455)
Supplement: S9 Methods — (DOCX) [file pmed.1003455.s009.docx]

**The potential shared role of inflammation in insulin resistance and schizophrenia: A bi-directional two-sample Mendelian randomization study**

Perry B.I. *et al*

**S9 Methods: SNPs used as instruments for glycated haemoglobin**

| rs1046896  rs10774625  rs11248914  rs11603334  rs11708067  rs11964178  rs12368284  rs9935401 | rs12621844  rs13134327  rs13266634  rs1387153  rs1547247  rs17509001  rs17533903 | rs17747324  rs1800562  rs2246434  rs2383208  rs267738  rs2979422  rs3782123 | rs423117  rs4607517  rs4737009  rs4745982  rs560887  rs579459  rs592423 | rs6474359  rs7040409  rs7616006  rs8192675  rs855791  rs9818758  rs9914988 |
| --- | --- | --- | --- | --- |
