# Supplementary material for: The potential shared role of inflammation in insulin resistance and schizophrenia: A bidirectional two-sample mendelian randomization study
Source: PLoS Med. 2021 Mar 12;18(3):e1003455. doi: 10.1371/journal.pmed.1003455 (PMC7954314; doi:10.1371/journal.pmed.1003455)
Supplement: S10 Methods — (DOCX) [file pmed.1003455.s010.docx]

**The potential shared role of inflammation in insulin resistance and schizophrenia: A bi-directional two-sample Mendelian randomization study**

Perry B.I. *et al*

| \| rs900400 \| \| --- \| \| rs6071166 \| | \| rs6738627 \| \| --- \| \| rs780093 \| |
| --- | --- | --- | --- | --- | --- |

**S10 Methods: SNPs used as instruments for leptin**
