# Supplementary material for: The potential shared role of inflammation in insulin resistance and schizophrenia: A bidirectional two-sample mendelian randomization study
Source: PLoS Med. 2021 Mar 12;18(3):e1003455. doi: 10.1371/journal.pmed.1003455 (PMC7954314; doi:10.1371/journal.pmed.1003455)
Supplement: S11 Methods — (DOCX) [file pmed.1003455.s011.docx]

**The potential shared role of inflammation in insulin resistance and schizophrenia: A bi-directional two-sample Mendelian randomization study**

Perry B.I. *et al*

**S11 Methods: MR Analysis Methods**

**Inverse Weighted Variance (IVW) Method**

IVW consists of a weighted linear regression of SNP-exposure SNP-outcome effect estimates. The IVW estimate is the inverse variance weighted mean of ratio estimates from 2 or more instruments [1], and assumes that all SNPs are valid instruments or that the sum of directional bias is zero. Since the intercept is an estimate of average pleiotropic effects across IVs, in an IVW approach the intercept is fixed to 0.

**Weighted Median**

The weighted median is the median of the weighted empirical distribution function of individual SNP ratio estimates. This method provides a consistent effect estimate if more than 50% of the information comes from valid SNPs [2].

**MR Egger**

MR-Egger regression consists of a weighted linear regression similar to IVW, with the assumption that horizontal pleiotropic effects and SNP-exposure associations are uncorrelated (i.e. the InSIDE assumption is not violated) [3], therefore the intercept is not fixed. MR Egger regression provides a valid effect estimate even if all SNPs are invalid instruments but assumes that uncertainty in the SNP-exposure association estimates is negligible (the ‘NOME’ assumption [4]).

**Mendelian Randomization Pleiotropy Residual Sum and Outlier (MR-PRESSO) Test**

MR-PRESSO [5] relies on a regression framework where the variants’ effects on the outcome are regressed on the same variants’ effects on exposure, with the slope of the regression line providing an estimate of the causal effect of the exposure on the outcome. The MR-PRESSO global test evaluates overall horizontal pleiotropy amongst all IVs in a single MR test by comparing the observed distance of all the variants to the regression line (residual sum of squares) to the expected distance under the null hypothesis of no horizontal pleiotropy. The MR-PRESSO outlier test evaluates the presence of specific horizontal pleiotropic outlier variants by using the observed and expected distributions of the tested variant. Finally, the MR-PRESSO distortion test evaluates the significance of the distortion between the causal estimate before and after removal of the horizontal pleiotropic outlier variants (detected from the outlier test of MR-PRESSO).

**Reference**

1. Burgess S, Butterworth A, Thompson SG. Mendelian randomization analysis with multiple genetic variants using summarized data. Genet Epidemiol. 2013;37(7):658-65.

2. Bowden J, Davey Smith G, Haycock PC, Burgess S. Consistent Estimation in Mendelian Randomization with Some Invalid Instruments Using a Weighted Median Estimator. Genet Epidemiol. 2016;40(4):304-14.

3. Bowden J, Davey Smith G, Burgess S. Mendelian randomization with invalid instruments: effect estimation and bias detection through Egger regression. Int J Epidemiol. 2015;44(2):512-25.

4. Bowden J, Del Greco MF, Minelli C, Davey Smith G, Sheehan N, Thompson J. A framework for the investigation of pleiotropy in two-sample summary data Mendelian randomization. Stat Med. 2017;36(11):1783-802.

5. Verbanck M, Chen CY, Neale B, Do R. Detection of widespread horizontal pleiotropy in causal relationships inferred from Mendelian randomization between complex traits and diseases. Nat Genet. 2018;50(5):693-8.
