# Supplementary material for: The potential shared role of inflammation in insulin resistance and schizophrenia: A bidirectional two-sample mendelian randomization study
Source: PLoS Med. 2021 Mar 12;18(3):e1003455. doi: 10.1371/journal.pmed.1003455 (PMC7954314; doi:10.1371/journal.pmed.1003455)
Supplement: S12 Methods — (DOCX) [file pmed.1003455.s012.docx]

**The potential shared role of inflammation in insulin resistance and schizophrenia: A bi-directional two-sample Mendelian randomization study**

Perry B.I. *et al*

**S12 Methods: Inflammation-related SNPs for fasting insulin, triglycerides and high-density lipoprotein**

**Fasting Insulin**

| **SNP** | **Inflammation-Related Pleiotropy** | **Effect Allele** |
| --- | --- | --- |
| rs2126259^a^ | CRP, Neutrophil Count, Granulocyte Count, Basophil Count, Myeloid White Cell Count | T |
| rs731839 | Lymphocyte Count, White Blood Cell Count, Neutrophil Count | G |
| rs10195252 | Lymphocyte Count, Neutrophil % of White Cells, | T |
| rs308971 | T-Cell Surface Protein CD3 Epsilon Chain | G |
| rs3822072 | CD32, Lymphocyte Count, Neutrophil Count | A |

^a^Genome-Wide Significance Inflammation-Related SNP; CRP=C-reactive protein

| **SNP** | **Inflammation-Related Pleiotropy** | **Effect Allele** |
| --- | --- | --- |
| rs10195252 | Lymphocyte Count, Neutrophil % of White cells, | T |
| rs731839 | Lymphocyte Count, White Blood Cell Count, Neutrophil Count | G |
| rs1011685 | Eosinophil Count, Granulocyte Count | C |
| rs3861397 | Eosinophil % Granulocytes, C-X-C Motif Chemokine 14 | G |

**Triglycerides**

| **SNP** | **Inflammation-Related Pleiotropy** | **Effect Allele** |
| --- | --- | --- |
| rs2126259^a^ | CRP, Neutrophil Count, Granulocyte Count, Basophil Count, Myeloid White Cell Count | T |
| rs731839 | Lymphocyte Count, White Blood Cell Count, Neutrophil Count | G |
| rs1011685 | Eosinophil % of White Cells, Neutrophil % of Granulocytes, Eosinophil Count | C |
| rs3822072 | CD32, Lymphocyte Count, Neutrophil Count | A |
| rs10195252 | Lymphocyte Count, Neutrophil % of White cells, | T |
| rs3861397 | Eosinophil % Granulocytes, C-X-C Motif Chemokine 14 | G |
| rs2745353 | Lymphocyte Count | T |

**HDL**^a^Genome-Wide Significance Inflammation-Related SNP
