# Supplementary material for: The potential shared role of inflammation in insulin resistance and schizophrenia: A bidirectional two-sample mendelian randomization study
Source: PLoS Med. 2021 Mar 12;18(3):e1003455. doi: 10.1371/journal.pmed.1003455 (PMC7954314; doi:10.1371/journal.pmed.1003455)
Supplement: S13 Methods — (DOCX) [file pmed.1003455.s013.docx]

**The potential shared role of inflammation in insulin resistance and schizophrenia: A bi-directional two-sample Mendelian randomization study**

Perry B.I. *et al*

**S13 Methods: Inflammation-related SNPs for low density lipoprotein**

| **SNP** | **Inflammation-Related Pleiotropy** | **Effect Allele** |
| --- | --- | --- |
| rs1169288^a^ | CRP | C |
| rs17404153^a^ | C-C Motif Chemokine 21 | G |
| rs174583^a^ | Neutrophil Count, Basophil Count, White Cell Count, Eosinophil Count | C |
| rs1800961^a^ | CRP, Neutrophil Count, Basophil Count | C |
| rs2642438 ^a^ | Granulocyte% White Cells Monocyte% White Cells | G |
| rs2886232 ^a^ | C-C Motif Chemokine 22 | T |
| rs2954029 ^a^ | Neutrophil Count, Eosinophil Count | A |
| rs3184504 ^a^ | Eosinophil Count, Basophil Count, Lymphocyte Count, WCC, Neutrophil Count, IL-2b | C |
| rs4970712 ^a^ | Monocyte Count, Granulocyte Count, Lymphocyte Count | C |
| rs579459 ^a^ | IL-3a, Neutrophil Count, Eosinophil Count, WCC, IL-6 | C |
| rs646776 ^a^ | CRP | T |
| rs9875338 ^a^ | Monocyte Count, Neutrophil Count | G |
| rs9987289 ^a^ | CRP, Neutrophil Count, Basophil Count | G |
| rs964184 | Eosinophil Count, Neutrophil% Granulocytes | G |
| rs75687619 | CRP | T |
| rs4253776 | Eosinophil% White Cells, Eosinophil% Granulocytes | G |
| rs2228603 | CRP, Lymphocyte Count | C |
| rs1408272 | Monocyte Count | T |
| rs10195252 | Lymphocyte Count, Neutrophil Count | T |
| rs12748152 | Basophil Count, Eosinophil Count, Eosinophil% Granulocytes, Neutrophil% Granulocytes | T |
| rs2000999 | Granulocyte% White Cells | A |
| rs2737252 | Monocyte Count | G |
| rs314253 | CD4:%Act(DR+38+) | T |
| rs676388 | Basophil Count | C |
| rs6882076 | Lymphocyte Count, WCC | C |

^a^Genome-Wide Significant Inflammation-Related SNP; CRP=C-reactive protein; WCC=White Cell Count; IL-=interleukin-.
