# Supplementary material for: The potential shared role of inflammation in insulin resistance and schizophrenia: A bidirectional two-sample mendelian randomization study
Source: PLoS Med. 2021 Mar 12;18(3):e1003455. doi: 10.1371/journal.pmed.1003455 (PMC7954314; doi:10.1371/journal.pmed.1003455)
Supplement: S14 Methods — (DOCX) [file pmed.1003455.s014.docx]

**The potential shared role of inflammation in insulin resistance and schizophrenia: A bi-directional two-sample Mendelian randomization study**

Perry B.I. *et al*

**S14 Methods: Inflammation-related SNPs for fasting plasma glucose**

| **SNP** | **Inflammation-Related Pleiotropy** | **Effect Allele** |
| --- | --- | --- |
| rs780093^a^ | Monocyte Count, Basophils, Neutrophil% | T |
| rs983309^a^ | Neutrophil%, Granulocytes | T |
| rs1130391138 | Lymphocyte% White Cells | A |
| rs2524299 | Basophil Count, Neutrophil Count, Granulocyte Count % White Cells, White Cell Count, Eosinophil Count, Monocyte Count | A |

^a^Genome-Wide Significant Inflammation-Related SNPs
