# Supplementary material for: The potential shared role of inflammation in insulin resistance and schizophrenia: A bidirectional two-sample mendelian randomization study
Source: PLoS Med. 2021 Mar 12;18(3):e1003455. doi: 10.1371/journal.pmed.1003455 (PMC7954314; doi:10.1371/journal.pmed.1003455)
Supplement: S15 Methods — (DOCX) [file pmed.1003455.s015.docx]

**The potential shared role of inflammation in insulin resistance and schizophrenia: A bi-directional two-sample Mendelian randomization study**

Perry B.I. *et al*

**S15 Methods: Inflammation-related SNPs for glycated haemoglobin**

| **SNP** | **Inflammation-Related Pleiotropy** | **Effect Allele** |
| --- | --- | --- |
| rs10774625^a^ | Neutrophil Count, Eosinophil Count, Monocyte Count, Lymphocyte Count, Basophil Count | A |
| rs11964178^a^ | Granulocyte% White Cells, Basophil Count, Neutrophil Count, White Cell Count, Lymphocyte Count | A |
| rs1547247 ^a^ | White Cell Count, Monocyte Count, Neutrophil Count, Granulocyte% White Cells | A |
| rs17509001^a^ | Lymphocyte Count, Monocyte Count, Neutrophil Count | C |
| rs4737009^a^ | Lymphocyte Count, Neutrophil Count | A |
| rs579459^a^ | IL-6, Neutrophil Count, Basophil Count, CRP | C |
| rs6474359^a^ | Lymphocyte Count | C |
| rs7616006 | White Cell Count, Monocyte Count, Lymphocyte Count, Neutrophil Count | A |
| rs1800562 | Monocyte Count | A |
| rs2246434 | Lymphocyte Count, Neutrophil Count, Lymphocyte% White Cells, Monocyte Count | A |

^a^Genome-Wide Significant Inflammation-Related SNP; CRP=C-reactive protein; IL-=interleukiin
