# Supplementary material for: The potential shared role of inflammation in insulin resistance and schizophrenia: A bidirectional two-sample mendelian randomization study
Source: PLoS Med. 2021 Mar 12;18(3):e1003455. doi: 10.1371/journal.pmed.1003455 (PMC7954314; doi:10.1371/journal.pmed.1003455)
Supplement: S16 Methods — (DOCX) [file pmed.1003455.s016.docx]

**The potential shared role of inflammation in insulin resistance and schizophrenia: A bi-directional two-sample Mendelian randomization study**

Perry B.I. *et al*

**S16 Methods: Inflammation-related SNPs for type 2 diabetes mellitus**

| **SNP** | **Inflammation-Related Pleiotropy** | **Effect Allele** |
| --- | --- | --- |
| rs1260326^a^ | CRP, Basophil Count, Neutrophil Count, Lymphocyte Count | C |
| rs2276853^a^ | Lymphocyte Count, Neutrophil Count, Granulocyte Count | A |
| rs2073721^a^ | Neutrophil Count, Monocyte %, Granulocyte % | G |
| rs1169288^a^ | CRP | C |
| rs1060105^a^ | Neutrophil Count, Lymphocyte Count | C |
| rs9891146^a^ | Neutrophil Count, Eosinophil Count, Granulocyte Count | T |
| rs1800961^a^ | CRP, Neutrophil Count, Granulocyte Count, Basophil Count, Myeloid Count | T |
| rs665268 | Monocyte Count | G |
| rs1801282 | Eosinophil% White Cells, Eosinophil Count | C |
| rs60980157 | Basophil Count, Neutrophil Count; Myeloid White Cell Count, White Cell Count | C |

^a^Genome-Wide Significant Inflammation-Related SNPs; CRP=C-reactive protein
