# Supplementary material for: The potential shared role of inflammation in insulin resistance and schizophrenia: A bidirectional two-sample mendelian randomization study
Source: PLoS Med. 2021 Mar 12;18(3):e1003455. doi: 10.1371/journal.pmed.1003455 (PMC7954314; doi:10.1371/journal.pmed.1003455)
Supplement: S17 Methods — (DOCX) [file pmed.1003455.s017.docx]

**The potential shared role of inflammation in insulin resistance and schizophrenia: A bi-directional two-sample Mendelian randomization study**

Perry B.I. *et al*

**S17 Methods: Inflammation-related SNPs for body mass index**

| **SNP** | **Inflammation-Related Pleiotropy** | **Effect Allele** |
| --- | --- | --- |
| rs16851483^a^ | Monocyte count | G |
| rs891389^a^ | Neutrophil %, Eosinophils, Basophils | C |
| rs1558902^a^ | CRP | A |
| rs571312^a^ | CRP | A |
| rs1000940 | Lymphocyte Count, White Cell Count | G |
| rs11663558 | Neutrophil Count, Granulocyte Count, Basophil Count, Myeloid White Cell Count | A |
| rs12448257 | Basophil Count, Neutrophil Count, Granulocyte Count, Myeloid White Cell Count, Eosinophil Count | G |
| rs13107325 | Eosinophil Count, Monocyte Count | C |
| rs205262 | Lymphocyte Count | A |
| rs3817334 | Granulocyte% Myeloid White Cells | C |
| rs4889606 | Lymphocyte Count | G |
| rs6567160 | Neutrophil Count, Myeloid Count, Granulocyte Count, White Cell Count | C |

^a^Genome-Wide Significant Inflammation-Related SNPs; CRP=C-reactive protein
