# Supplementary material for: The potential shared role of inflammation in insulin resistance and schizophrenia: A bidirectional two-sample mendelian randomization study
Source: PLoS Med. 2021 Mar 12;18(3):e1003455. doi: 10.1371/journal.pmed.1003455 (PMC7954314; doi:10.1371/journal.pmed.1003455)
Supplement: S18 Methods — (DOCX) [file pmed.1003455.s018.docx]

**The potential shared role of inflammation in insulin resistance and schizophrenia: A bi-directional two-sample Mendelian randomization study**

Perry B.I. *et al*

**S18 Methods: Inflammation-related SNPs for schizophrenia**

| **SNP** | **Inflammation-Related Pleiotropy** | **Effect Allele** |
| --- | --- | --- |
| rs2851447 | Lymphocyte%, Neutrophil% | G |
| rs3130820 | Lymphocyte Count, Basophil Count, Monocyte count | T |
| rs4925114 | Lymphocyte Count | T |
| rs12416331 | Monocyte Count | A |
| rs7216638 | White cell count, Basophil count | T |
