# Supplementary material for: The potential shared role of inflammation in insulin resistance and schizophrenia: A bidirectional two-sample mendelian randomization study
Source: PLoS Med. 2021 Mar 12;18(3):e1003455. doi: 10.1371/journal.pmed.1003455 (PMC7954314; doi:10.1371/journal.pmed.1003455)
Supplement: S19 Methods — (DOCX) [file pmed.1003455.s019.docx]

**The potential shared role of inflammation in insulin resistance and schizophrenia: A bi-directional two-sample Mendelian randomization study**

Perry B.I. *et al*

**S19 Methods: SNPs used for CRP in MVMR Analysis**

| rs1205* |
| --- |
| rs3093077 |
| rs1130864* |
| rs1800947 |

*SNPs pruned during clumping procedure
