# Supplementary material for: The potential shared role of inflammation in insulin resistance and schizophrenia: A bidirectional two-sample mendelian randomization study
Source: PLoS Med. 2021 Mar 12;18(3):e1003455. doi: 10.1371/journal.pmed.1003455 (PMC7954314; doi:10.1371/journal.pmed.1003455)
Supplement: S1 Results — (DOCX) [file pmed.1003455.s020.docx]

**The potential shared role of inflammation in insulin resistance and schizophrenia: A bi-directional two-sample Mendelian randomization study**

Perry B.I. *et al*

**S1 Results: Multivariable MR (MVMR) Results^a^ for IR-Phenotype Exposures (All-SNP analysis) with Addition of CRP as Exposure**

| **Risk Factor** | **no. SNPs** | **Odds Ratio (95% C.I.)** | **P-value** |
| --- | --- | --- | --- |
| Fasting Insulin | 10 | 0.96 (0.66-1.38) | 0.813 |
| *CRP* | *2* | *0.88 (0.62-1.23)^b^* | *0.456* |
| Triglycerides | 10 | 0.98 (0.88-1.10) | 0.756 |
| *CRP* | *2* | *1.00 (0.0.65-1.56)^b^* | *0.987* |
| HDL | 15 | 1.00 (0.86-1.18) | 0.937 |
| *CRP* | *2* | *0.92 (0.71-1.76)^b^* | *0.489* |

CRP=C-reactive protein; HDL=high-density lipoprotein; SNPs=single nucleotide polymorphisms

^a^Results for IVW MVMR analysis
^b^We did not perform univariable MR analysis for CRP since this was not a goal of the study. Univariable MR has been conducted and replicated for CRP and estimates are published elsewhere [1, 2]

**Reference**

1. Lin BD, Alkema A, Peters T, Zinkstok J, Libuda L, Hebebrand J, et al. Assessing causal links between metabolic traits, inflammation and schizophrenia: a univariable and multivariable, bidirectional Mendelian-randomization study. Int J Epidemiol. 2019;48(5):1505-14.

2. Hartwig FP, Borges MC, Horta BL, Bowden J, Davey Smith G. Inflammatory Biomarkers and Risk of Schizophrenia: A 2-Sample Mendelian Randomization Study. JAMA Psychiatry. 2017;74(12):1226-33.
