# Supplementary material for: The potential shared role of inflammation in insulin resistance and schizophrenia: A bidirectional two-sample mendelian randomization study
Source: PLoS Med. 2021 Mar 12;18(3):e1003455. doi: 10.1371/journal.pmed.1003455 (PMC7954314; doi:10.1371/journal.pmed.1003455)
Supplement: S2 Results — (DOCX) [file pmed.1003455.s021.docx]

**The potential shared role of inflammation in insulin resistance and schizophrenia: A bi-directional two-sample Mendelian randomization study**

Perry B.I. *et al*

**S2 Results: Multivariable MR (MVMR) Results^a^ for IR-Phenotype Exposures (Inflammation-related-SNP analysis) with Addition of CRP as Exposure**

| **Risk Factor** | **Genome-Wide Significant Inflammation-Related SNPs** | | | **Nominally Significant Inflammation-Related SNPs** | | |
| --- | --- | --- | --- | --- | --- | --- |
|  | **no. SNPs** | **Odds Ratio (95% C.I.)** | ***P*-value** | **no. SNPs** | **Odds Ratio (95% CI)** | ***P*-value** |
| Fasting Insulin | 1 | 1.02 (0.37-2.78) | 0.975 | 5 | 1.46 (0.85-2.51) | 0.307 |
| CRP | *2* | 0.94 (0.40-2.18)*^b^* | 0.881 | 2 | 1.27 (0.80-2.02) | 0.308 |
| Triglycerides | - | - | - | 4 | 1.06 (0.91-1.25) | 0.447 |
| CRP | *-* | - | - | 2 | 0.70 (0.45-1.45) | 0.343 |
| HDL | 1 | 1.00 (0.85-1.16) | 0.849 | 7 | 0.99 (0.81-1.21) | 0.731 |
| CRP | *2* | 0.90 (0.72-1.12)*^b^* | 0.367 | 2 | 0.90 (0.76-1.08) | 0.251 |

CRP=C-reactive protein; HDL=high-density lipoprotein; SNPs=single nucleotide polymorphisms

^a^Results for IVW MVMR analysis

^b^We did not perform univariable MR analysis for CRP since this was not a goal of the study. Univariable MR has been conducted and replicated for CRP and estimates are published elsewhere [1, 2]

**Reference**

1. Lin BD, Alkema A, Peters T, Zinkstok J, Libuda L, Hebebrand J, et al. Assessing causal links between metabolic traits, inflammation and schizophrenia: a univariable and multivariable, bidirectional Mendelian-randomization study. Int J Epidemiol. 2019;48(5):1505-14.

2. Hartwig FP, Borges MC, Horta BL, Bowden J, Davey Smith G. Inflammatory Biomarkers and Risk of Schizophrenia: A 2-Sample Mendelian Randomization Study. JAMA Psychiatry. 2017;74(12):1226-33.
