# Supplementary material for: The potential shared role of inflammation in insulin resistance and schizophrenia: A bidirectional two-sample mendelian randomization study
Source: PLoS Med. 2021 Mar 12;18(3):e1003455. doi: 10.1371/journal.pmed.1003455 (PMC7954314; doi:10.1371/journal.pmed.1003455)
Supplement: S3 Results — (DOCX) [file pmed.1003455.s022.docx]

**The potential shared role of inflammation in insulin resistance and schizophrenia: A bi-directional two-sample Mendelian randomization study**

Perry B.I. *et al*

**S3 Results: MR Analyses using all SNPs for Schizophrenia and Cardiometabolic Outcomes**

| **Outcome** | **SNPs, No.** | **Method** | **β (S.E)** | **P-value** | **Corrected *p*-value^a^** |
| --- | --- | --- | --- | --- | --- |
| Fasting Insulin | 101 | IVW | 0.01 (0.02) | 0.496 | 1.000 |
|  |  | Weighted Median | 0.02 (0.02) | 0.268 | 1.000 |
|  |  | MR Egger | -0.05 (0.08) | 0.542 | 1.000 |
| Triglycerides | 101 | IVW | 0.00 (0.02) | 0.970 | 1.000 |
|  |  | Weighted Median | 0.00 (0.03) | 0.987 | 1.000 |
|  |  | MR Egger | 0.05 (0.11) | 0.642 | 1.000 |
| HDL | 101 | IVW | -0.02 (0.03) | 0.521 | 1.000 |
|  |  | Weighted Median | -0.01 (0.03) | 0.901 | 1.000 |
|  |  | MR Egger | -0.04 (0.05) | 0.051 | 0.510 |
| Fasting Plasma Glucose | 105 | IVW | 0.01 (0.01) | 0.339 | 1.000 |
|  |  | Weighted Median | 0.01 (0.01) | 0.454 | 1.000 |
|  |  | MR Egger | 0.00 (0.06) | 0.994 | 1.000 |
| Type 2 Diabetes Mellitus | 109 | IVW | -0.01 (0.06) | 0.845 | 1.000 |
|  |  | Weighted Median | 0.00 (0.08) | 1.000 | 1.000 |
|  |  | MR Egger | 0.14 (0.30) | 0.645 | 1.000 |
| Body Mass Index | 101 | IVW | -0.03 (0.02) | 0.220 | 1.000 |
|  |  | Weighted Median | -0.03 (0.02) | 0.146 | 1.000 |
|  |  | MR Egger | 0.18 (0.10) | 0.081 | 0.729 |
| HbA1C | 104 | IVW | 0.01 (0.01) | 0.911 | 1.000 |
|  |  | Weighted Median | 0.01 (0.02) | 0.730 | 1.000 |
|  |  | MR Egger | 0.01 (0.07) | 0.948 | 1.000 |
| Glucose Tolerance | 101 | IVW | 0.08 (0.07) | 0.278 | 1.000 |
|  |  | Weighted Median | 0.12 (0.10) | 0.233 | 1.000 |
|  |  | MR Egger | 0.24 (0.35) | 0.496 | 1.000 |
| LDL | 101 | IVW | -0.06 (0.03) | 0.079 | 0.790 |
|  |  | Weighted Median | -0.06 (0.05) | 0.080 | 0.800 |
|  |  | MR Egger | -0.22 (0.14) | 0.113 | 0.904 |
| Leptin | 101 | IVW | 0.02 (0.02) | 0.239 | 1.000 |
|  |  | Weighted Median | 0.01 (0.03) | 0.677 | 1.000 |
|  |  | MR Egger | -0.02 (0.09) | 0.810 | 1.000 |

HDL=high-density lipoprotein; HbA1C=glycated haemoglobin; LDL=low-density lipoprotein; SNPs=single nucleotide polymorphisms; IVW=inverse variance weighted regression; β=beta coefficient; S.E=standard error. ^a^Adjusted using the Holm-Bonferroni method for multiple testing.
