# Supplementary material for: The potential shared role of inflammation in insulin resistance and schizophrenia: A bidirectional two-sample mendelian randomization study
Source: PLoS Med. 2021 Mar 12;18(3):e1003455. doi: 10.1371/journal.pmed.1003455 (PMC7954314; doi:10.1371/journal.pmed.1003455)
Supplement: S4 Results — (DOCX) [file pmed.1003455.s023.docx]

**The potential shared role of inflammation in insulin resistance and schizophrenia: A bi-directional two-sample Mendelian randomization study**

Perry B.I. *et al*

**S4 Results: The Association between Inflammation-Related Schizophrenia SNPs and Cardiometabolic Outcomes**

| **Outcome** | **No. immune SNPs** | **Method** | **β (S.E)** | **P-value** | **Corrected P-value^a^** |
| --- | --- | --- | --- | --- | --- |
| Fasting Insulin | 3 | IVW | 0.04 (0.05) | 0.409 | 1.000 |
|  |  | Weighted Median | 0.03 (0.06) | 0.666 | 1.000 |
|  |  | MR Egger | 0.00 (0.09) | 0.976 | 1.000 |
| Triglycerides | 3 | IVW | 0.20 (0.09) | 0.034 | 0.340 |
|  |  | Weighted Median | 0.20 (0.08) | 0.009 | 0.090 |
|  |  | MR Egger | 0.28 (0.30) | 0.306 | 1.000 |
| HDL | 1 | Wald Ratio | -0.26 (0.21) | 0.202 | 1.000 |
| LDL | 3 | IVW | 0.11 (0.07) | 0.953 | 1.000 |
|  |  | Weighted Median | 0.06 (0.07) | 0.341 | 1.000 |
|  |  | MR Egger | -0.01 (0.10) | 0.895 | 1.000 |
| Body Mass Index | 4 | IVW | -0.02 (0.09) | 0.750 | 1.000 |
|  |  | Weighted Median | 0.00 (0.05) | 0.832 | 1.000 |
|  |  | MR Egger | 0.05 (0.02) | 0.705 | 1.000 |
| Type 2 Diabetes Mellitus | 3 | IVW | -0.18 (0.34) | 0.598 | 1.000 |
|  |  | Weighted Median | 0.10 (0.30) | 0.729 | 1.000 |
|  |  | MR Egger | 0.24 (0.80) | 0.789 | 1.000 |
| Fasting Plasma Glucose | 4 | IVW | -0.02 (0.07) | 0.780 | 1.000 |
|  |  | Weighted Median | -0.05 (0.04) | 0.317 | 1.000 |
|  |  | MR Egger | -0.16 (0.10) | 0.258 | 1.000 |
| HbA1C | 3 | IVW | -0.07 (0.07) | 0.269 | 1.000 |
|  |  | Weighted Median | 0.06 (0.05) | 0.137 | 1.000 |
|  |  | MR Egger | -0.18 (0.12) | 0.292 | 1.000 |
| Glucose Tolerance | 4 | IVW | 0.12 (0.26) | 0.648 | 1.000 |
|  |  | Weighted Median | 0.10 (0.31) | 0.732 | 1.000 |
|  |  | MR Egger | -0.09 (0.49) | 0.872 | 1.000 |
| Leptin | 4 | IVW | -0.03 (0.07) | 0.646 | 1.000 |
|  |  | Weighted Median | -0.04 (0.08) | 0.619 | 1.000 |
|  |  | MR Egger | -0.10 (0.12) | 0.526 | 1.000 |

HDL=high-density lipoprotein; HbA1C=glycated haemoglobin; LDL=low-density lipoprotein; SNPs=single nucleotide polymorphisms; IVW=inverse variance weighted regression; β=beta coefficient; S.E=standard error. ^a^Adjusted using the Holm-Bonferroni method for multiple testing.
