# Supplementary material for: The potential shared role of inflammation in insulin resistance and schizophrenia: A bidirectional two-sample mendelian randomization study
Source: PLoS Med. 2021 Mar 12;18(3):e1003455. doi: 10.1371/journal.pmed.1003455 (PMC7954314; doi:10.1371/journal.pmed.1003455)
Supplement: S5 Results — (DOCX) [file pmed.1003455.s024.docx]

**The potential shared role of inflammation in insulin resistance and schizophrenia: A bi-directional two-sample Mendelian randomization study**

Perry B.I. *et al*

**S5 Results: Cochran’s Q Tests for Heterogeneity and MR Egger Intercept Tests for Horizontal Pleiotropy for the Association between all Cardiometabolic SNPs and Schizophrenia**

|  | **IVW** | | **MR Egger** | | | |
| --- | --- | --- | --- | --- | --- | --- |
| **Cardiometabolic Risk Factor** | **Cochran’s Q (df)** | ***p-*value** | **Cochran’s Q (df)** | ***p-*value** | **MR Egger Intercept (SE)** | **Intercept *p-*value** |
| Fasting Insulin | 19.37 (8) | 0.013 | 11.03 (7) | 0.137 | -0.05 (0.02) | 0.055 |
| Triglycerides | 46.66 (9) | <0.001 | 40.88 (8) | <0.001 | -0.01 (0.01) | 0.319 |
| HDL | 65.05 (14) | <0.001 | 46.80 (13) | <0.001 | 0.02 (0.01) | 0.032 |
| Fasting Plasma Glucose | 50.11 (21) | <0.001 | 49.90 (20) | <0.001 | <0.01 (0.01) | 0.773 |
| Type 2 Diabetes Mellitus | 119.81 (26) | <0.001 | 118.68 (25) | <0.001 | -0.01 (0.01 | 0.646 |
| Body Mass Index | 320.28 (81) | <0.001 | 304.74 (80) | <0.001 | -0.01 (0.01) | 0.047 |
| HbA1C | 66.04 (34) | 0.008 | 63.05 (33) | 0.001 | -0.01 (0.00) | 0.219 |
| Glucose Tolerance | 14.47 (6) | 0.024 | 7.47 (5) | 0.188 | -0.06 (0.03) | 0.083 |
| Leptin | 10.45 (3) | 0.015 | 6.75 (2) | 0.034 | -0.12 (0.11) | 0.405 |
| LDL | 141.44 (75) | <0.001 | 141.39 (74) | <0.001 | <0.01 (0.00) | 0.873 |

IVW=inverse variance weighted regression; df=degrees of freedom; SE=standard error; HDL=high-density lipoprotein; HbA1C=glycated haemoglobin; LDL=low-density lipoprotein.
