# Supplementary material for: The potential shared role of inflammation in insulin resistance and schizophrenia: A bidirectional two-sample mendelian randomization study
Source: PLoS Med. 2021 Mar 12;18(3):e1003455. doi: 10.1371/journal.pmed.1003455 (PMC7954314; doi:10.1371/journal.pmed.1003455)
Supplement: S6 Results — (DOCX) [file pmed.1003455.s025.docx]

**The potential shared role of inflammation in insulin resistance and schizophrenia: A bi-directional two-sample Mendelian randomization study**

Perry B.I. *et al*

**S6 Results: Cochran’s Q Tests for Heterogeneity and MR Egger Intercept Tests for Horizontal Pleiotropy for the Association between Inflammation-Related Cardiometabolic SNPs and Schizophrenia**

|  | **IVW** | | **MR Egger** | | | |
| --- | --- | --- | --- | --- | --- | --- |
| **Cardiometabolic Risk Factor** | **Cochran’s Q (df)** | ***p-*value** | **Cochran’s Q (df)** | ***p-*value** | **Regression Intercept (SE)** | **Direction *p-*value** |
| Fasting Insulin | * | * | * | * | * | * |
| HDL | * | * | * | * | * | * |
| Type 2 Diabetes Mellitus | 34.89 (6) | <0.001 | 32.71 (5) | <0.001 | -0.02 (0.03) | 0.589 |
| Fasting Plasma Glucose | 7.09 (1) | 0.008 | * | * | * | * |
| HbA1C | 34.89 (6) | <0.001 | 32.71 (5) | <0.001 | -0.01 (0.01) | 0.628 |
| Body Mass Index | 1.51 (4) | 0.471 | 1.00 (3) | 0.752 | 0.02 (0.02) | 0.446 |
| LDL | 37.29 (11) | <0.001 | 32.65 (10) | 0.001 | 0.01 (0.01) | 0.261 |

IVW=inverse variance weighted regression; df=degrees of freedom; SE=standard error; HDL=high-density lipoprotein; HbA1C=glycated haemoglobin; LDL=low-density lipoprotein.
*insufficient *n* SNPs
