# Supplementary material for: The potential shared role of inflammation in insulin resistance and schizophrenia: A bidirectional two-sample mendelian randomization study
Source: PLoS Med. 2021 Mar 12;18(3):e1003455. doi: 10.1371/journal.pmed.1003455 (PMC7954314; doi:10.1371/journal.pmed.1003455)
Supplement: S7 Results — (DOCX) [file pmed.1003455.s026.docx]

**The potential shared role of inflammation in insulin resistance and schizophrenia: A bi-directional two-sample Mendelian randomization study**

Perry B.I. *et al*

**S7 Results: Cochran’s Q Tests for Heterogeneity and MR Egger Intercept Tests for Horizontal Pleiotropy for the Association between Schizophrenia SNPs and Cardiometabolic Outcomes**

|  | **IVW** | | **MR Egger** | | | |
| --- | --- | --- | --- | --- | --- | --- |
| **Cardiometabolic Outcome** | **Cochran’s Q (df)** | ***p-*value** | **Cochran’s Q (df)** | ***p-*value** | **Regression Intercept (SE)** | **Direction *p-*value** |
| Fasting Insulin | 129.93 (100) | 0.024 | 128.96 (99) | 0.023 | 0.00 (0.00) | 0.442 |
| Triglycerides | 205.86 (100) | <0.001 | 205.37 (99) | <0.001 | 0.00 (0.00) | 0.628 |
| HDL | 373.38 (100) | <0.001 | 353.54 (99) | <0.001 | 0.01 (0.00) | 0.020 |
| Fasting Plasma Glucose | 125.03 (104) | 0.078 | 124.98 (103) | 0.069 | 0.00 (0.00) | 0.843 |
| Type 2 Diabetes Mellitus | 139.83 (108) | 0.021 | 139.50 (107) | 0.019 | 0.00 (0.01) | 0.612 |
| Body Mass Index | 264.27 (100) | <0.001 | 264.80 (99) | <0.001 | -0.01 (0.00) | 0.041 |
| HbA1C | 131.35 (103) | 0.031 | 131.34 (102) | 0.027 | 0.00 (0.00) | 0.966 |
| Glucose Tolerance | 110.78 (100) | 0.217 | 110.54 (99) | 0.201 | -0.01 (0.01) | 0.642 |
| Leptin | 6.76 (2) | 0.034 | 0.388 (1) | 0.533 | -0.16 (0.06) | 0.240 |
| LDL | 183.06 (100) | <0.001 | 175.38 (99) | <0.001 | 0.01 (0.00) | 0.040 |

IVW=inverse variance weighted regression; df=degrees of freedom; SE=standard error; HDL=high-density lipoprotein; HbA1C=glycated haemoglobin; LDL=low-density lipoprotein.
