# Supplementary material for: The potential shared role of inflammation in insulin resistance and schizophrenia: A bidirectional two-sample mendelian randomization study
Source: PLoS Med. 2021 Mar 12;18(3):e1003455. doi: 10.1371/journal.pmed.1003455 (PMC7954314; doi:10.1371/journal.pmed.1003455)
Supplement: S8 Results — (DOCX) [file pmed.1003455.s027.docx]

**The potential shared role of inflammation in insulin resistance and schizophrenia: A bi-directional two-sample Mendelian randomization study**

Perry B.I. *et al*

**S8 Results: Cochran’s Q Tests for Heterogeneity and MR Egger Intercept Tests for Horizontal Pleiotropy for the Association between Inflammation-Related Schizophrenia SNPs and Cardiometabolic Outcomes**

| **Cardiometabolic Outcome** | **IVW** | | **MR Egger** | | | |
| --- | --- | --- | --- | --- | --- | --- |
|  | **Cochran’s Q (df)** | ***p*-value** | **Cochran’s Q (df)** | ***p-*value** | **Regression Intercept (SE)** | **Direction *p-*value** |
| Fasting Insulin | 6.83 (5) | 0.233 | 6.50 (4) | 0.165 | 0.00 (0.00) | 0.676 |
| Triglycerides | 73.73 (5) | <0.001 | 56.79 (4) | <0.001 | -0.02 (0.02) | 0.336 |
| HDL | 4.00 (1) | 0.050 | * | * | * | * |
| Type 2 Diabetes Mellitus | 13.30 (5) | 0.021 | 11.10 (4) | 0.026 | 0.02 (0.03) | 0.421 |
| Fasting Plasma Glucose | 6.91 (3) | 0.075 | 2.97 (2) | 0.226 | 0.01 (0.01) | 0.245 |
| Body Mass Index | 36.18 (5) | <0.001 | 25.96 (4) | <0.001 | -0.01 (0.01) | 0.278 |
| LDL | 4.36 (2) | 0.113 | 0.16 (1) | 0.687 | 0.01 (0.00) | 0.289 |
| HbA1C | 6.24 (3) | 0.100 | 4.21 (2) | 0.121 | 0.00 (0.00) | 0.430 |
| Glucose Tolerance | 0.80 (2) | 0.671 | 0.787 (1) | 0.375 | 0.00 (0.03) | 0.941 |
| Leptin | 0.42 (2) | 0.812 | 0.351 (1) | 0.554 | 0.00 (0.00) | 0.841 |

IVW=inverse variance weighted regression; df=degrees of freedom; SE=standard error; HDL=high-density lipoprotein; HbA1C=glycated haemoglobin; LDL=low-density lipoprotein.
*insufficient *n* SNPs
