# Supplementary material for: The potential shared role of inflammation in insulin resistance and schizophrenia: A bidirectional two-sample mendelian randomization study
Source: PLoS Med. 2021 Mar 12;18(3):e1003455. doi: 10.1371/journal.pmed.1003455 (PMC7954314; doi:10.1371/journal.pmed.1003455)
Supplement: S9 Results — (DOCX) [file pmed.1003455.s028.docx]

**The potential shared role of inflammation in insulin resistance and schizophrenia: A bi-directional two-sample Mendelian randomization study**

Perry B.I. *et al*

**S9 Results: MR-PRESSO Tests of Cardiometabolic All-SNP Analysis to Examine For and Correct Horizontal Pleiotropy**

| **Risk Factor** | **MR-PRESSO Global Test** | | **Outlier-Corrected IVW** | | **Distortion Test** | |
| --- | --- | --- | --- | --- | --- | --- |
|  | **RSS** | ***p*-value** | **β (SE)** | ***p*-value** | **Coefficient** | ***p*-value** |
| Fasting Insulin | 24.35 | 0.018 | 0.08 (0.18) | 0.669 | 171.67 | 0.156 |
| Triglycerides | 71.43 | <0.001 | 0.23 (0.06) | 0.008 | -64.56 | 0.531 |
| HDL | 85.02 | <0.001 | -0.12 (0.08) | 0.169 | 92.64 | 0.666 |
| Fasting Plasma Glucose | 53.78 | <0.001 | 0.03 (0.05) | 0.594 | 120.88 | 0.300 |
| Type 2 Diabetes Mellitus | 148.58 | <0.001 | -0.06 (0.06) | 0.390 | -54.32 | 0.353 |
| Body Mass Index | 328.03 | <0.001 | 0.02 (0.07) | 0.815 | 187.40 | 0.255 |
| HbA1C | 69.33 | 0.002 | 0.06 (0.12) | 0.651 | -93.64 | 0.828 |
| Glucose Tolerance | 20.77 | 0.020 | * | * | * | * |
| LDL | 148.15 | <0.001 | -0.01 (0.03) | 0.581 | 46.44 | 0.840 |
| Leptin | 32.73 | 0.002 | 0.27 (0.25) | 0.382 | 22.42 | 0.338 |

MR PRESSO= Mendelian Randomization Pleiotropy Residual Sum and Outlier; β=beta coefficient; S.E=standard error. IVW=inverse variance weighted regression; df=degrees of freedom; SE=standard error; HDL=high-density lipoprotein; HbA1C=glycated haemoglobin; LDL=low-density lipoprotein.
*no evidence of horizontal pleiotropy
