# Supplementary material for: The potential shared role of inflammation in insulin resistance and schizophrenia: A bidirectional two-sample mendelian randomization study
Source: PLoS Med. 2021 Mar 12;18(3):e1003455. doi: 10.1371/journal.pmed.1003455 (PMC7954314; doi:10.1371/journal.pmed.1003455)
Supplement: S10 Results — (DOCX) [file pmed.1003455.s029.docx]

**The potential shared role of inflammation in insulin resistance and schizophrenia: A bi-directional two-sample Mendelian randomization study**

Perry B.I. *et al*

**S10 Results: MR-PRESSO Tests of Inflammation-Related Cardiometabolic SNPs to Examine For and Correct Horizontal Pleiotropy**

| **Risk Factor** | **MR-PRESSO Global Test** | | **Outlier-Corrected IVW** | | **Distortion Test** | |
| --- | --- | --- | --- | --- | --- | --- |
|  | **RSS** | ***p*-value** | **β (SE)** | ***p*-value** | **Coefficient** | ***p*-value** |
| Fasting Insulin | † | † | † | † | † | † |
| HDL | † | † | † | † | † | † |
| Fasting Plasma Glucose | † | † | † | † | † | † |
| Type 2 Diabetes Mellitus | 47.76 | 0.001 | 0.11 (0.13) | 0.436 | -162.05 | 0.210 |
| Body Mass Index | 12.93 | 0.124 | † | † | † | † |
| HbA1C | 10.24 | 0.409 | * | * | * | * |
| LDL | 50.16 | 0.001 | 0.00 (0.07) | 0.968 | -261.84 | 0.230 |

MR PRESSO= Mendelian Randomization Pleiotropy Residual Sum and Outlier; β=beta coefficient; S.E=standard error. IVW=inverse variance weighted regression; df=degrees of freedom; SE=standard error; HDL=high-density lipoprotein; HbA1C=glycated haemoglobin; LDL=low-density lipoprotein.
*no evidence of horizontal pleiotropy; †no identified outliers
