# Supplementary material for: The potential shared role of inflammation in insulin resistance and schizophrenia: A bidirectional two-sample mendelian randomization study
Source: PLoS Med. 2021 Mar 12;18(3):e1003455. doi: 10.1371/journal.pmed.1003455 (PMC7954314; doi:10.1371/journal.pmed.1003455)
Supplement: S11 Results — (DOCX) [file pmed.1003455.s030.docx]

**The potential shared role of inflammation in insulin resistance and schizophrenia: A bi-directional two-sample Mendelian randomization study**

Perry B.I. *et al*

**S11 Results: MR-PRESSO Tests of Schizophrenia All-SNP Analysis to Examine For and Correct Horizontal Pleiotropy**

| **Outcome** | **MR-PRESSO Global Test** | | **Outlier-Corrected IVW** | | **Distortion Test** | |
| --- | --- | --- | --- | --- | --- | --- |
|  | **RSS** | ***p*-value** | **β (SE)** | ***p*-value** | **Coefficient** | ***p*-value** |
| Fasting Insulin | 161.53 | 0.020 | † | † | † | † |
| Triglycerides | 249.82 | <0.001 | 0.00 (0.02) | 0.210 | 590.84 | 0.064 |
| HDL | 434.93 | <0.001 | -0.01 (-0.02) | 0.567 | 117.13 | 0.251 |
| Fasting Plasma Glucose | 155.12 | 0.067 | * | * | * | * |
| Type 2 Diabetes Mellitus | 174.18 | 0.012 | † | † | † | † |
| Body Mass Index | 372.10 | <0.001 | -0.04 (0.02) | 0.014 | 1.89 | 0.966 |
| HbA1C | 149.23 | 0.107 | * | * | * | * |
| Glucose Tolerance | 137.63 | 0.235 | * | * | * | * |
| LDL | 216.08 | <0.001 | 0.00 (0.02) | 0.866 | -501.07 | 0.100 |
| Leptin | 113.87 | 0.772 | * | * | * | * |

MR PRESSO= Mendelian Randomization Pleiotropy Residual Sum and Outlier; β=beta coefficient; S.E=standard error. IVW=inverse variance weighted regression; df=degrees of freedom; SE=standard error; HDL=high-density lipoprotein; HbA1C=glycated haemoglobin; LDL=low-density lipoprotein.
*no evidence of horizontal pleiotropy; †no identified outliers
