# Supplementary material for: The potential shared role of inflammation in insulin resistance and schizophrenia: A bidirectional two-sample mendelian randomization study
Source: PLoS Med. 2021 Mar 12;18(3):e1003455. doi: 10.1371/journal.pmed.1003455 (PMC7954314; doi:10.1371/journal.pmed.1003455)
Supplement: S12 Results — (DOCX) [file pmed.1003455.s031.docx]

**The potential shared role of inflammation in insulin resistance and schizophrenia: A bi-directional two-sample Mendelian randomization study**

Perry B.I. *et al*

**S12 Results: MR-PRESSO Tests of Inflammation-Related Schizophrenia SNP Analysis to Examine For and Correct Horizontal Pleiotropy**

| **Outcome** | **MR-PRESSO Global Test** | | **Outlier-Corrected IVW** | | **Distortion Test** | |
| --- | --- | --- | --- | --- | --- | --- |
|  | **RSS** | ***p*-value** | **β (SE)** | ***p*-value** | **Coefficient** | ***p*-value** |
| Fasting Insulin | 1.08 | 0.883 | * | * | * | * |
| Triglycerides | 23.51 | 0.058 | * | * | * | * |
| HDL | 9.56 | 0.276 | * | * | * | * |
| Fasting Plasma Glucose | 15.34 | 0.095 | * | * | * | * |
| Type 2 Diabetes Mellitus | 18.41 | 0.048 | 0.22 (0.09) | 0.144 | -182.77 | <0.001 |
| Body Mass Index | 15.11 | 0.128 | * | * | * | * |
| HbA1C | 10.81 | 0.182 | * | * | * | * |
| Glucose Tolerance | 2.54 | 0.729 | * | * | * | * |
| LDL | 14.13 | 0.165 | * | * | * | * |
| Leptin | 3.29 | 0.647 | * | * | * | * |

MR PRESSO= Mendelian Randomization Pleiotropy Residual Sum and Outlier; β=beta coefficient; S.E=standard error. IVW=inverse variance weighted regression; df=degrees of freedom; SE=standard error; HDL=high-density lipoprotein; HbA1C=glycated haemoglobin; LDL=low-density lipoprotein.
*no evidence of horizontal pleiotropy
