# Supplementary material for: The potential shared role of inflammation in insulin resistance and schizophrenia: A bidirectional two-sample mendelian randomization study
Source: PLoS Med. 2021 Mar 12;18(3):e1003455. doi: 10.1371/journal.pmed.1003455 (PMC7954314; doi:10.1371/journal.pmed.1003455)
Supplement: S13 Results — (DOCX) [file pmed.1003455.s032.docx]

**The potential shared role of inflammation in insulin resistance and schizophrenia: A bi-directional two-sample Mendelian randomization study**

Perry B.I. *et al*

**S13 Results: I_2GX_ Statistics to Examine for Potential Violation of the ‘No Measurement Error’ (NOME) Assumption for MR Egger Analyses**

| **Exposure** | **I^2^_GX_ of SNP-Exposure Associations** | |
| --- | --- | --- |
|  | **All-SNP Analyses** | **Inflammation-Related SNP Analyses** |
| Fasting Insulin | 0.95 | * |
| Triglycerides | 0.99 | * |
| HDL | 0.99 | * |
| Fasting Plasma Glucose | 0.95 | 0.90 |
| Type 2 Diabetes Mellitus | 0.84 | 0.62 |
| Body Mass Index | 0.93 | 0.99 |
| HbA1C | 0.94 | 0.91 |
| Glucose Tolerance | 0.51 | * |
| Leptin | 0.00 | * |
| LDL | 0.99 | 0.98 |
| Schizophrenia | 0.00 | 0.83 |

HDL=high-density lipoprotein; HbA1C=glycated haemoglobin; LDL=low-density lipoprotein.
*insufficient *n* SNPs
