# Supplementary material for: The potential shared role of inflammation in insulin resistance and schizophrenia: A bidirectional two-sample mendelian randomization study
Source: PLoS Med. 2021 Mar 12;18(3):e1003455. doi: 10.1371/journal.pmed.1003455 (PMC7954314; doi:10.1371/journal.pmed.1003455)
Supplement: S1 Checklist — (DOCX) [file pmed.1003455.s033.docx]

**The potential shared role of inflammation in insulin resistance and schizophrenia: A bi-directional two-sample Mendelian randomization study**

Perry B.I. *et al*

**S1 Checklist: STROBE-MR: Guidelines for strengthening the reporting of Mendelian randomization studies [1]**

**1. TITLE and ABSTRACT**

Indicate Mendelian randomization as the study’s design in the title and/or the abstract.
Title; Abstract – Methods & Findings.

# INTRODUCTION 2. Background

Explain the scientific background and rationale for the reported study. Is causality between exposure and outcome plausible? Justify why MR is a helpful method to address the study question.
Introduction – Paragraphs 1-2.

**3. Objectives**

State specific objectives clearly, including pre-specified causal hypotheses (if any).
Introduction – Paragraph 4.

# METHODS

# 4. Study design and data sources

Present key elements of study design early in the paper. Consider including a table listing sources of data for all phases of the study.
Methods – Paragraph 1; S1 Methods.

# 5. Assumptions

Explicitly state assumptions for the main analysis (e.g. relevance, exclusion, independence, homogeneity) as well assumptions for any additional or sensitivity analysis.
Methods – Statistical Analysis – Paragraph 1-2; S10 Methods.

# 6. Statistical methods: main analysis

Describe statistical methods and statistics used.

1. Describe how quantitative variables were handled in the analyses (i.e., scale, units, model).
   Methods – Statistical Analysis – Paragraph 1.
2. Describe the process for identifying genetic variants and weights to be included in the analyses (i.e, independence and model)
   Methods – Statistical Analysis – Paragraph 1.
3. Describe the MR estimator, e.g. two-stage least squares, Wald ratio, and related statistics. Detail the included covariates and, in case of two-sample MR, whether the same covariate set was used for adjustment in the two samples.
   Methods – Statistical Analysis – Paragraph 1; S10 Methods.
4. If applicable, say how multiple testing was dealt with.
   Methods – Correction for Multiple Testing – Paragraph 1.

1. **Assessment of assumptions**

Describe any methods used to assess the assumptions or justify their validity.
Methods – Statistical Analysis – Paragraph 2.

1. **Sensitivity analyses**

Describe any sensitivity analyses or additional analyses performed.
Methods – Statistical Analysis – Paragraph 2; Methods – Analysis using Inflammation-Related SNPs – Paragraphs 1-2; Methods – Sensitivity Analysis – Adjustment for Inflammation – Paragraph 1.

# 9. Software and pre-registration

1. Name statistical software and package(s), including version and settings used.
   Methods – Statistical Analysis – Paragraph 1.
2. State whether the study protocol and details were pre-registered (as well as when and where).
   Methods – Statistical Analysis – Paragraph 1.

# RESULTS 10. Descriptive data

1. For two-sample Mendelian randomization:

Provide information on extent of sample overlap between the exposure and outcome data sources.
Methods – Selection of Genetic Variants Related to Cardiometabolic Traits and Schizophrenia – Paragraph 1.

# 11. Main results

1. Report the associations between genetic variant and exposure, and between genetic variant and outcome, preferably on an interpretable scale (e.g. comparing 25^th^ and 75^th^ percentile of allele count or genetic risk score, if individual-level data available).
   Figure 1, Figure 2, Figure S1.
2. Report causal effect estimate between exposure and outcome, and the measures of uncertainty from the MR analysis. Use an intuitive scale, such as odds ratio, or relative risk, per standard deviation difference.
   Results Table 1; Results Table 2; S1 Results; S2 Results; S3 Results; S4 Results.
3. If relevant, consider translating estimates of relative risk into absolute risk for a meaningful time-period.
   N/A.
4. Consider any plots to visualize results (e.g. forest plot, scatterplot of associations between genetic variants and outcome versus between genetic variants and exposure).
   Figure 1, Figure 2, Figure S1.

# 12. Assessment of assumptions

1. Assess the validity of the assumptions.
   Results – Test for Horizontal Pleiotropy – Paragraphs 1&2; Results – Test for Heterogeneity of Instruments – Paragraph 1; Results – Test for Measurement Error – Paragraph 1.
2. Report any additional statistics (e.g., assessments of heterogeneity, such as I^2^, Q statistic).
   S5-S13 Results.

# 13. Sensitivity and additional analyses

1. Use sensitivity analyses to assess the robustness of the main results to violations of the assumptions.
   S5-S13 Results.
2. Report results from other sensitivity analyses (e.g., replication study with different dataset, analyses of subgroups, validation of instrument(s), simulations, etc.)
   Results – MR Analyses using Inflammation-related Genetic Variants for IR and Other Cardiometabolic Traits – Paragraph 1; Results – Sensitivity Analysis: Adjustment for Inflammation – Paragraph 1; Table 2; S1-2 Results.
3. Report any assessment of direction of causality (e.g., bidirectional MR).
   Results – Test for Bidirectionality using Schizophrenia as Exposure – Paragraph 1.

**DISCUSSION 14. Key results**

Summarize key results with reference to study objectives.
Discussion – Paragraph 1.

# 15. Limitations

Discuss limitations of the study, taking into account the validity of the MR assumptions, other sources of potential bias, and imprecision. Discuss both direction and magnitude of any potential bias, and any efforts to address them.
Discussion – Strengths and Limitations – Paragraph 2.

# 16. Interpretation

1. Give a cautious overall interpretation of results considering objectives and limitations. Compare with results from other relevant studies.
   Discussion – Conclusion – Paragraph 1.
2. Discuss underlying biological mechanisms that could be modelled by using the genetic variants to assess the relationship between the exposure and the outcome.
   Discussion – Inflammation as a Common Cause for Schizophrenia and Insulin Resistance – Paragraphs 1-5.
3. Discuss whether the results have clinical or policy relevance, and whether interventions could have the same size effect.
   Discussion – Conclusion – Paragraph 1.

# 17. Generalizability

Discuss the generalizability of the study results (a) to other populations (i.e. external validity), (b) across other exposure periods/timings, and (c) across other levels of exposure.
Discussion – Paragraphs 1-10.

# OTHER INFORMATION 18. Funding

Give the source of funding and the role of the funders for the present study and, if applicable, for the original study or studies on which the present article is based.
Funding Statement

# 19. Data and data sharing

Present data used to perform all analyses or report where and how the data can be accessed. State whether statistical code is publicly accessible and if so, where.
Data Availability Statement

# 20. Conflicts of Interest

All authors should declare all potential conflicts of interest.
 Competing Interest Statement

**Reference**

1. Burgess S, Davey Smith, G., Davies, N.M., Dudbridge, F., Gill, D., Glymour, M.M., Hartwig, F.P., Holmes, M.V., Minelli, C., Relton, C.L., Theodoratou, E. Guidelines for performing Mendelian randomization investigations [version 2; peer review: 2 approved]. Wellcome Open Res. 2020;4(186).
