# Supplementary material for: The potential shared role of inflammation in insulin resistance and schizophrenia: A bidirectional two-sample mendelian randomization study
Source: PLoS Med. 2021 Mar 12;18(3):e1003455. doi: 10.1371/journal.pmed.1003455 (PMC7954314; doi:10.1371/journal.pmed.1003455)
Supplement: S2 Checklist — (DOCX) [file pmed.1003455.s034.docx]

**The potential shared role of inflammation in insulin resistance and schizophrenia: A bi-directional two-sample Mendelian randomization study**

Perry B.I. *et al*

**S2 Checklist: STROBE Statement [1]**

|  | Item No | Recommendation | Where Located |
| --- | --- | --- | --- |
| **Title and abstract** | 1 | (*a*) Indicate the study’s design with a commonly used term in the title or the abstract | Title |
|  |  | (*b*) Provide in the abstract an informative and balanced summary of what was done and what was found | Abstract – Methods & Findings |
| Introduction | | | |
| Background/rationale | 2 | Explain the scientific background and rationale for the investigation being reported | Introduction Paragraphs 1-3 |
| Objectives | 3 | State specific objectives, including any prespecified hypotheses | Introduction Paragraph 4 |
| Methods | | | |
| Study design | 4 | Present key elements of study design early in the paper | Introduction Paragraph 4; Methods – Statistical Analysis Paragraph 1; S10 Methods |
| Setting | 5 | Describe the setting, locations, and relevant dates, including periods of recruitment, exposure, follow-up, and data collection | Methods – Selection of Genetic Variants Related to Cardiometabolic Traits and Schizophrenia – Paragraph 1; S1-10 Methods |
| Participants | 6 | (*a*) *Cohort study*—Give the eligibility criteria, and the sources and methods of selection of participants. Describe methods of follow-up  *Case-control study*—Give the eligibility criteria, and the sources and methods of case ascertainment and control selection. Give the rationale for the choice of cases and controls  *Cross-sectional study*—Give the eligibility criteria, and the sources and methods of selection of participants | Methods – Selection of Genetic Variants Related to Cardiometabolic Traits and Schizophrenia – Paragraph 1; S1 Methods |
|  |  | (*b*) *Cohort study*—For matched studies, give matching criteria and number of exposed and unexposed  *Case-control study*—For matched studies, give matching criteria and the number of controls per case | Methods – Selection of Genetic Variants Related to Cardiometabolic Traits and Schizophrenia – Paragraph 1; S1 Methods |
| Variables | 7 | Clearly define all outcomes, exposures, predictors, potential confounders, and effect modifiers. Give diagnostic criteria, if applicable | Methods – Selection of Genetic Variants Related to Cardiometabolic Traits and Schizophrenia – Paragraph 1; S1 Methods |
| Data sources/ measurement | 8* | For each variable of interest, give sources of data and details of methods of assessment (measurement). Describe comparability of assessment methods if there is more than one group | Methods – Selection of Genetic Variants Related to Cardiometabolic Traits and Schizophrenia – Paragraph 1; S1-S9 Methods; Methods – Statistical Analysis – Parahraphs 1-2 |
| Bias | 9 | Describe any efforts to address potential sources of bias | Methods – Statistical Analysis – Paragraph 2 |
| Study size | 10 | Explain how the study size was arrived at | Methods – Selection of Genetic Variants Related to Cardiometabolic Traits and Schizophrenia – Paragraph 1; S1 Methods |
| Quantitative variables | 11 | Explain how quantitative variables were handled in the analyses. If applicable, describe which groupings were chosen and why | Methods – Statistical Analysis – Paragraph 1 |
| Statistical methods | 12 | (*a*) Describe all statistical methods, including those used to control for confounding | Methods – Statistical Analysis – Paragraphs 1-2; Methods - Statistical Analysis – Analysis using Inflammation-related SNPs; Methods – Statistical Analysis – Sensitivity Analysis – Adjustment for inflammation |
|  |  | (*b*) Describe any methods used to examine subgroups and interactions | Methods - Statistical Analysis – Analysis using Inflammation-related SNPs; Methods – Statistical Analysis – Sensitivity Analysis – Adjustment for inflammation |
|  |  | (*c*) Explain how missing data were addressed | N/A |
|  |  | (*d*) *Cohort study*—If applicable, explain how loss to follow-up was addressed  *Case-control study*—If applicable, explain how matching of cases and controls was addressed  *Cross-sectional study*—If applicable, describe analytical methods taking account of sampling strategy | N/A |
|  |  | (*e*) Describe any sensitivity analyses | Methods - Statistical Analysis – Analysis using Inflammation-related SNPs; Methods – Statistical Analysis – Sensitivity Analysis – Adjustment for inflammation |

Continued on next page

| Results | | | |
| --- | --- | --- | --- |
| Participants | 13* | (a) Report numbers of individuals at each stage of study—eg numbers potentially eligible, examined for eligibility, confirmed eligible, included in the study, completing follow-up, and analysed | Methods – Paragraph 1; S1 Methods; |
|  |  | (b) Give reasons for non-participation at each stage | N/A |
|  |  | (c) Consider use of a flow diagram | N/A |
| Descriptive data | 14* | (a) Give characteristics of study participants (eg demographic, clinical, social) and information on exposures and potential confounders | S1 Methods |
|  |  | (b) Indicate number of participants with missing data for each variable of interest | N/A |
|  |  | (c) *Cohort study*—Summarise follow-up time (eg, average and total amount) | N/A |
| Outcome data | 15* | *Cohort study*—Report numbers of outcome events or summary measures over time | N/A |
|  |  | *Case-control study—*Report numbers in each exposure category, or summary measures of exposure | Methods – Paragraph 1; S1 Methods; |
|  |  | *Cross-sectional study—*Report numbers of outcome events or summary measures | N/A |
| Main results | 16 | (*a*) Give unadjusted estimates and, if applicable, confounder-adjusted estimates and their precision (eg, 95% confidence interval). Make clear which confounders were adjusted for and why they were included | Results Table 1; Results Table 2; S1 Results; S2 Results; S3 Results; S4 Results. |
|  |  | (*b*) Report category boundaries when continuous variables were categorized | N/A |
|  |  | (*c*) If relevant, consider translating estimates of relative risk into absolute risk for a meaningful time period | N/A |
| Other analyses | 17 | Report other analyses done—eg analyses of subgroups and interactions, and sensitivity analyses | Results – MR Analyses using Inflammation-related Genetic Variants for IR and Other Cardiometabolic Traits – Paragraph 1; Results – Sensitivity Analysis: Adjustment for Inflammation – Paragraph 1; Table 2; S1-2 Results. |
| Discussion | | | |
| Key results | 18 | Summarise key results with reference to study objectives | Discussion – Main Findings - Paragraph 1 |
| Limitations | 19 | Discuss limitations of the study, taking into account sources of potential bias or imprecision. Discuss both direction and magnitude of any potential bias | Discussion – Strengths and Limitations – Paragraph 2 |
| Interpretation | 20 | Give a cautious overall interpretation of results considering objectives, limitations, multiplicity of analyses, results from similar studies, and other relevant evidence | Discussion – Main Findings – Paragraph 1; Discussion – Inflammation as a Common Cause for Schizophrenia and Insulin Resistance – Paragraphs 1-6; Discussion – Additional Findings – Paragraph 1 |
| Generalisability | 21 | Discuss the generalisability (external validity) of the study results | Discussion – Main Findings – Paragraph 1; Discussion – Inflammation as a Common Cause for Schizophrenia and Insulin Resistance – Paragraphs 1-6; Discussion – Additional Findings – Paragraph 1 |
| Other information | | | |
| Funding | 22 | Give the source of funding and the role of the funders for the present study and, if applicable, for the original study on which the present article is based | Funding Statement |

**Reference**

1. von Elm E, Altman DG, Egger M, Pocock SJ, Gotzsche PC, Vandenbroucke JP, et al. The Strengthening the Reporting of Observational Studies in Epidemiology (STROBE) statement: guidelines for reporting observational studies. PLoS Med. 2007;4(10):e296.
