# Supplementary material for: The potential shared role of inflammation in insulin resistance and schizophrenia: A bidirectional two-sample mendelian randomization study
Source: PLoS Med. 2021 Mar 12;18(3):e1003455. doi: 10.1371/journal.pmed.1003455 (PMC7954314; doi:10.1371/journal.pmed.1003455)
Supplement: S1 Fig — (DOCX) [file pmed.1003455.s035.docx]

**The potential shared role of inflammation in insulin resistance and schizophrenia: A bi-directional two-sample Mendelian randomization study**

Perry B.I. *et al*

**S1 Figure: Forest Plot Illustrating MR Analyses of Schizophrenia as Outcome using All SNPs (green) and Inflammation-Related SNPs (purple)**

**
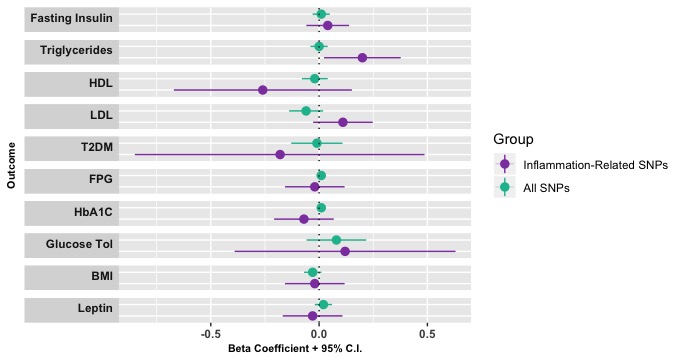
**Forest plot presents beta coefficients and 95% CIs for MR analyses between inflammation and non-inflammation-related schizophrenia SNPs and cardiometabolic outcomes. Results are presented for IVW analysis HDL=High Density Lipoprotein; T2DM=Type 2 Diabetes Mellitus; BMI=Body Mass Index; FPG=Fasting Plasma Glucose; LDL=Low-Density Lipoprotein; HbA1C=Glycated Haemoglobin; Glucose Tol=Glucose Tolerance. See S3 and S4 Results for full numerical results for the IVW analysis presented in this figure alongside other MR analysis methods.
