# Supplementary material for: The potential shared role of inflammation in insulin resistance and schizophrenia: A bidirectional two-sample mendelian randomization study
Source: PLoS Med. 2021 Mar 12;18(3):e1003455. doi: 10.1371/journal.pmed.1003455 (PMC7954314; doi:10.1371/journal.pmed.1003455)
Supplement: Attachment — Submitted filename: Response to Reviewer & Editor Comments.docx [file pmed.1003455.s036.docx]

**Evidence for Inflammation as a Putative Shared Mechanism for Insulin Resistance and Schizophrenia: A Mendelian Randomization Study**

Perry B.I. *et al*

**Response to Reviewer and Editor Comments**

**Editor:** Your paper was evaluated by a senior editor and discussed among all the editors here. It was also discussed with an academic editor with relevant expertise, and sent to three independent reviewers, including a statistical reviewer. The reviews are appended at the bottom of this email and any accompanying reviewer attachments can be seen via the link below.

**Reply:** We are grateful to the editors and reviewers for their time and efforts in appraising our manuscript. The comments received were of high quality and have helped us to clarify our findings and improve our manuscript. Below, we present a list of reviewer comments alongside our responses to each comment. We submit two versions of the amended manuscript; one clean copy and a second annotated copy with our changes highlighted. The annotated copy also includes line numbers, which we refer to in the comments below.

**Comments From The Editor**

**Editor:** 1) Please completely address the comments of reviewers 2 and 3, including clarification of the hypothesis put forth in the introduction, in particular regarding what is meant for inflammation being a “shared mechanism” behind cardiometabolic disorders and schizophrenia.  As requested by reviewers, please also include more of an explanation of the rationale and implications behind the different MR methods, with more clarity in the interpretation of the results of the different tests performed, as this is helpful to readers who may lack specific knowledge of the MR methods.

**Reply:** Please see below for our itemised responses to each comment raised by reviewers 2 and 3. We have endeavoured to address each point raised in sufficient detail and make changes to our work where necessary to fully address the points raised by reviewers. In addition, we have divided the discussion with subheadings to improve clarity.

**Editor:** 2.Competing Interests: Please add this statement to the manuscript's Competing Interests: "SB is a paid statistical consultant on PLOS Medicine's statistical board."  Please add this statement to the manuscript's Competing Interests: "CL is an Academic Editor on PLOS Medicine's editorial board."

**Reply:** We have added this information to the Competing Interests statement (P25 line 5).

**Editor:** 3. Prospective protocol: Did your study have a prospective protocol or analysis plan? Please state this (either way) early in the Methods section.
a) If a prospective analysis plan (from your funding proposal, IRB or other ethics committee submission, study protocol, or other planning document written before analyzing the data) was used in designing the study, please include the relevant prospectively written document with your revised manuscript as a Supporting Information file to be published alongside your study, and cite it in the Methods section. A legend for this file should be included at the end of your manuscript. 
b) If no such document exists, please make sure that the Methods section transparently describes when analyses were planned, and when/why any data-driven changes to analyses took place.  
c) In either case, changes in the analysis-- including those made in response to peer review comments-- should be identified as such in the Methods section of the paper, with rationale.

**Reply**: The analysis plan was not formally registered to a repository or database. As requested, we have clarified this in the methods (P9 line 7; P11 line 9). Plans for our primary analysis were formed before commencing the work after discussions among the study authors. The plan was informed by an *a priori* hypothesis also conceived by the study authors; that inflammation may be a common mechanism for insulin resistance and schizophrenia. Our primary analysis consisted of examining whether potential causal relationships may exist of insulin resistance (IR) and a set of related cardiometabolic traits with schizophrenia. Secondly, we examined inflammation-related variants for the cardiometabolic traits to test a hypothesis of inflammation as a common mechanism. In lieu of the findings from the primary analysis, we performed a *post-hoc* sensitivity analysis where we used multi-variable MR to examine whether the associations evident in the MR analysis of inflammatory-related variants could be explained by inflammation. We did this to check the stability of the findings from our primary analysis.

**Editor:** 4. Data Availability Statement: The Data Availability Statement (DAS) requires revision. You mention the sources of the summary statistics used in the manuscript.  For each data source used in your study please note here: 
a) If the data are freely or publicly available, note this and state the location of the data: within the paper, in Supporting Information files, or in a public repository (include the DOI or accession number).
b) If the data are owned by a third party but freely available upon request, please note this and state the owner of the data set and contact information for data requests (web or email address). Note that a study author cannot be the contact person for the data.

**Reply**: We have added a Data Availability Statement at the end of the manuscript, detailing the location & method of access for the GWAS summary statistics we used in our study (P25 line 10).

**Editor:** 5. Abstract: Background: The final sentence should state the goal or question of the study.

**Reply:** We have added a sentence at the end of the abstract background providing the aims of the study “We aimed to examine whether insulin resistance and seven related cardiometabolic traits may be causally associated with schizophrenia, and whether inflammation may be a common mechanism for cardiometabolic disorders and schizophrenia”.

**Editor:** 6. Abstract: Methods and Findings: For the “large genome-wide association studies” please provide some detail describing the demographics of the participants of those studies, the names of the studies/cohorts.

**Reply:** We have provided further detail in the “Methods and Findings” section of the abstract as suggested.

**Editor:** 7. Abstract: Methods and Findings: Line 21-22, and throughout: please reduce the use of causal language- we suggest: “Our study found no evidence in support of a causal relationship between schizophrenia and insulin resistance.” or similar.  Please similarly adjust causal language throughout the manuscript.

**Reply**: We have made the suggested change to the abstract and have made amendments to the reporting of the results in the main manuscript to reduce the use of causal language (P12 line 4 onward).

**Editor**: 8. Abstract: Please report effects with p values and 95% CIs when describing the main results of the study (associations between factors and schizophrenia individually and the multivariable MR findings with CRP).

**Reply**: We have amended the abstract to include corrected *p*-values alongside full results for the MVMR analyses.

**Editor:** 9. Abstract: Methods and Findings: In the last sentence of the Abstract Methods and Findings section, please describe the main limitation(s) of the study's methodology.

**Reply:** We have added a sentence describing a primary limitation of the study methodology in the abstract. We now state “A potential limitation of the study is that the full range of gene-products from the genetic variants we used as proxies for the exposures is unknown, and so we are unable to comment on potential biological mechanisms of association other than inflammation, which may also be relevant.”

**Editor:** 10. Abstract: Conclusions: Please address the study implications without overreaching what can be concluded from the data; how do the results described in the Methods and Findings speak to the mechanism where treating inflammation may prevent cardiometabolic disease in schizophrenia?

**Reply:** We have amended our abstract conclusion and specifically mention that “inflammation may be a common cause for schizophrenia and cardiometabolic disorders, which may at least partly explain why they commonly co-occur in clinical practice”. Therefore, “Inflammation and immune pathways may represent novel therapeutic targets for the prevention or treatment of schizophrenia and cardiometabolic disorders”, and “Future work is needed to understand how inflammation simultaneously increases the risk of schizophrenia and cardiometabolic disorders”.

**Editor:** 11. Author Summary: At this stage, we ask that you include a short, non-technical Author Summary of your research to make findings accessible to a wide audience that includes both scientists and non-scientists. The Author Summary should immediately follow the Abstract in your revised manuscript. This text is subject to editorial change and should be distinct from the scientific abstract. Please see our author guidelines for more information: https://journals.plos.org/plosmedicine/s/revising-your-manuscript#loc-author-summary

**Reply:** We have added an author summary (P4 line 1).

**Editor:** 12. Methods: Please provide the name(s) of the institutional review board(s) that provided ethical approval.

**Reply:** Our study reports a secondary analysis of existing publicly available datasets, therefore formal IRB approval of this project was not required. All included data were obtained from GWAS which had appropriate ethical approval in place. We have clarified this within the text (P8 line 20).

**Editor:** 13. Methods: Please provide summary demographic information from the GWAS cohorts (e.g. population, setting, relevant characteristics).

**Reply:** We have provided further demographic information within S1 Methods, including population, setting and relevant characteristics. We have also added a footnote to the table directing readers to the referenced GWAS studies for detailed information on the included studies.

**Editor:** 14. Results: First paragraph: “The strength of evidence for these associations weakened substantially after correction for multiple testing”  Please provide the OR, 95% CIs and p values for the findings corrected for multiple testing here.  Similarly, in the paragraph describing the findings for IR-related SNPs, please provide the results when corrected for multiple testing.

**Reply:** We have redrafted the first paragraph in the results to clarify that evidence did not surpass the corrected p-value threshold, and have amended the results to present p-values after correcting for multiple testing (P12 line 4). In the paragraph reporting results for inflammation-related SNPs, we now present the p-values after correction for multiple testing (P14 line 3).

**Editor:** 15. Results: Top of page 17: “Test for Bidirectionality using Schizophrenia as Exposure”: In this section, please revise to say, “We did not find a statistically significant MR association between…” or similar language throughout this paragraph.  Similarly, please revise the first sentence of the next section “Test for horizontal pleiotropy”

**Reply:** We have amended the language of this section as suggested (P16 line 28; P17 line 5).

**Editor:** 16. Discussion: Second sentence of first paragraph: Please revise this sentence, and similar sentences throughout the text to make it clear that such associations did not reach the significance level that was adjusted for multiple comparisons, in addition to being inconsistent across MR methods.  “In analyses using all associated SNPs, we report some evidence that genetically predicted levels of triglycerides, HDL and leptin may be associated with schizophrenia, although estimates were inconsistent across MR methods and may have been affected by horizontal pleiotropy.”

**Reply:** We have made amendments to ensure discussion of the results of the all-SNP analysis makes clear that the evidence did not survive correction for multiple testing (P19 line 8; P19 line 20; P21 line 9).

**Editor:** 17. References: Please be sure to use the "Vancouver" style for reference formatting (including in the supplementary files), and see our website for other reference guidelines: https://journals.plos.org/plosmedicine/s/submission-guidelines#loc-references

**Reply:** We have made the change to ensure referencing in the main manuscript file and supplementary data files match PLOS Medicine formatting guidelines.

**Editor:** 18. Table 1 and Table 2: Please define all abbreviations found within the tables in the legends.

**Reply:** We have added a footnote to define abbreviations in both tables.

**Editor:** 19. Figure 1: In the legend, please define abbreviations for IVW MR, SNPs, HDL, and please describe the nature of the points, whiskers and lines in the plots.  Please increase the size of the fonts used.

**Reply:** We have redrafted the footnote from Figure 1 to describe in more detail the nature of the points, whiskers and lines in plots, as well as define abbreviations (P15 line 13).

**Editor:** 20. Figure 2: Please define abbreviations for SNP in the legend.

**Reply:** We have included a definition for the abbreviation for SNP in the footnote (P16 line 3).

**Editor:** 21. Figure 3: Please define abbreviations for MVMR, and CRP, in the legend.

**Reply:** We have updated the footnote of Figure 3 with definitions of abbreviations used (P16 line 18).

**Editor:** 22. Supporting information tables and figures: Please provide legends for each individual table and figure in the Supporting Information.  In the legends, please define each abbreviation that appears in the corresponding figure/table.  Within the manuscript please refer to individual items as “S13 Table” to clarify to which supporting information item you are referring.

**Reply:** We have added footnotes for each individual table and figure within the supporting information. We have amended the names of supporting information tables/figures to match the descriptions within the text.

**Editor:** 23. Checklist: Please ensure that the study is reported according to the STROBE guideline, and include the completed STROBE checklist as Supporting Information. When completing the checklist, please use section and paragraph numbers, rather than page numbers. Please add the following statement, or similar, to the Methods: "This study is reported as per the Strengthening the Reporting of Observational Studies in Epidemiology (STROBE) guideline (S1 Checklist)."

**Reply:** We agree with the editor that aligning to formal reporting guidelines is preferential. Recently, a new reporting guideline has been produced (STROBE-MR) (Davey Smith et al, 2019; Burgess et al, 2020) specifically for the reporting of MR studies. We have included a completed checklist for STROBE-MR in our supporting information (S1 Checklist), which is referenced within the text (P10 line 11).

**Comments From The Reviewers**

**Reviewer #1**

**Reviewer:** Thank you for the opportunity to review this paper. It was a pleasure to read. The authors did an excellent job in presenting the background and rationale for the study as well as a complex series of analyses is a succinct fashion. The results are novel and important both for clinicians and researchers.

**Reply:** We thank the reviewer for their kind appraisal of our work.

**Reviewer #2**

**Reviewer:** The aim of this study is to investigate whether insulin resistance is a causal risk factor for schizophrenia using publicly available summary genetic data and a two-sample Mendelian randomization (MR) design. Further to this, a secondary objective of the paper is to study if inflammation is a potential mechanism explaining this association. The research question is interesting and two-sample MR is emerging as a gold standard approach (despite it is not without limitations) to ask causal questions. However, I have a number of concerns regarding this study, mainly about how the authors appraised the existing evidence and interpreted their results, and the methods used to explore the question under study. Below, I highlight a number of major and minor concerns.

**Reply:** We thank the reviewer for their time and efforts appraising our work, and for the detailed and helpful comments which have allowed us to clarify and improve our manuscript.

**Reviewer:** Major concerns 1. A more careful critical appraisal of the literature is required throughout the manuscript. 
1a. Page 4, lines 13-19. The evidence presented here is largely cross-sectional, but the authors appear to draw causal inferences from it ("therefore, IR, a significant risk factors for T2DM and obesity, and schizophrenia, may share pathophysiologic mechanism"). The authors should explicitly mention the risk of reverse causality here. Also, they reference a longitudinal study (Perry et al. 2018) which finds no evidence of a longitudinal association of IR and psychotic experiences, thus strengthening evidence of reverse causality.

**Reply:** We agree with the reviewer that a greater understanding of the relationship between cardiometabolic dysfunction and schizophrenia is hampered by the cross-sectional nature of most existing research. We agree that findings from longitudinal research are relatively scarce. As the reviewer correctly states, in our own previous study, we found evidence of a cross-sectional association between insulin resistance (IR) at age 18 and risk of psychosis, but we did not find longitudinal evidence for an association between IR at age 9 and risk of psychosis at age 18 (Perry et al, 2018). The cross-sectional and heterogenous nature of the majority of previous research was a major factor in our choice of method to examine the research question; we performed a bidirectional MR analysis which allowed us to examine direction of association and also address problems of residual confounding. We have clarified this section of the introduction to make clear that previous research in the field is mostly cross-sectional (P6 line 17) and that reverse causality is possible in light of longitudinal findings (P6 line 21).

**Reviewer:** 1b. Reverse causality is mentioned on page 4, lines 21-23 but in relation to 'observational evidence', here it should be made clear that this statement refers to  cross-sectional observational evidence, not all observational evidence.

**Reply:** We have clarified that specifically cross-sectional research is limited by reverse causality (P6 line 17).

**Reviewer:** 1c. Page 4, lines 23-25 the authors should make it clear that residual confounding represents a separate issue that affects cross-sectional and longitudinal evidence.

**Reply:** We have made the clarification that residual confounding is an issue that affects both cross-sectional and longitudinal research (P6 line 22).

**Reviewer:** 1d.  Overall, it would benefit the introduction to more clearly explain why in the absence of longitudinal evidence on the association between IR and schizophrenia, and the authors' previous evidence that IR does not mediate IL6/psychotic experiences findings, a causal role of the former and a mechanistic role of inflammation was hypothesized.

**Reply:** We thank the reviewer for the opportunity to clarify. Our proposed hypotheses were: (1) insulin resistance is causally associated with schizophrenia; and, (2) inflammation may be a common mechanism for insulin resistance and schizophrenia. These hypotheses were derived in light of the following evidence (see below). We have now made the rationale for our work clearer in the introduction (P7 line 16).

a) Consistent reports from previous research (meta-analyses of case-control studies) showing associations between IR and first-episode psychosis in relatively young adults.
b) Consistent reports from previous research (meta-analyses of cross-sectional studies; longitudinal studies; MR studies) showing associations between inflammation and psychosis
c) Consistent reports from previous research (cross-sectional studies, longitudinal studies) showing associations between inflammation and IR

The reviewer is correct that in a previous observational study cited in the text, we did not find evidence of mediation between IL-6 at age 9 and risk of psychosis at age 18 (Perry et al, 2018). However, mediation is not the only means by which a role of inflammation may exist in the association between IR and schizophrenia. Please see our response to the comment below (point 2b) where we discuss the possible nature of the mechanistic link and how we tested it, in greater detail.

**Reviewer:** 1e. The study cited on page 20 line 23 (reference 44) is based on 22 cases of schizophrenia and the study did not adjust for stressful life events, existing mental and physical illness and other important stressors that could confound the association between CRP and schizophrenia. These limitations should be noted, as at the moment the article is presented uncritically as providing evidence for CRP as a risk factor for schizophrenia.

**Reply**: We agree with the reviewer that there are limitations to the observational reports regarding CRP and schizophrenia. We have clarified this within the text (P20 line 18). However, we do not aim to suggest that CRP is a risk factor for SCZ, and we do not aim to test this in our analysis. Instead, we used CRP in as a general downstream marker of systemic inflammation. We have now clarified this point in the paragraph noted by the reviewer (P20 line 15). Our analyses first tested MR associations of IR and related cardiometabolic traits with schizophrenia (and *vice versa* in bidirectional analysis) using all SNPs related to relevant cardiometabolic trait. Then, to explore role of inflammation, we used a subset of genetic variants for cardiometabolic traits that were also associated with inflammation as instruments in MR analysis. These variants were chosen based on their associations with a range of immune markers and not CRP specifically. Following this, to confirm that associations for inflammation-related genetic variants were indeed likely driven by inflammation, we used CRP in multivariable MR to examine whether the associations of inflammatory-related cardiometabolic variants attenuated after adjustment for CRP. The observed attenuation confirms the role of inflammation in the relationship. Hence, our aim for using CRP was to confirm the role of inflammation in general, and not to suggest that CRP is a causal risk factor for schizophrenia.

**Reviewer:** 2.  Methodological concerns:  a.  In lines 9-14 on page 5, the authors say that both CRP and IL6 have been shown to be associated with schizophrenia in both cohort studies, and MR analyses. However, evidence for the association between CRP and psychotic experiences from observational studies is a lot more tenuous than that for IL6. Similarly, in MR analyses lower CRP and higher IL6 are associated with schizophrenia (mentioned in the discussion). The authors should discuss this more clearly and should more explicitly explain the rationale for using CRP as opposed to IL6 in these analyses in light of this evidence.

**Reply:** As described in response to previous comment, we used CRP in multi-variable MR analysis to explore whether the estimates from the analysis examining the association of inflammatory-related cardiometabolic genetic variants with schizophrenia were likely driven by inflammation. We used CRP because it is a common downstream marker of systemic inflammation (as we mention on P8 line 6), and crucially, has been studied in large-scale GWAS for which summary data are publicly available. We did not hypothesise a specific role for CRP per se in the association between IR and schizophrenia. We have amended our text to make this point clear (P11 line 13; P20 line 14).

We agree with the reviewer that we could have selected other immune/inflammatory measures in place of CRP, such as IL-6, for example. However, the CRP data is based on an exceptionally large sample, and similarly scaled publicly available summary data for other inflammatory markers are scarce (as we have clarified on P21 line 4).

**Reviewer:** b. Lines 23-24 page 5, the authors say that they investigated whether inflammation could be a shared mechanism between IR and schizophrenia. However, it is not clear what is meant by that, e.g. does inflammation predispose to both IR and SCZ? Or does IR lead to inflammation which then leads to SCZ? More clarity around the hypothesized mechanisms is needed.

**Reply:** We are grateful to the reviewer for the opportunity to clarify and expand upon the response to the comment raised above (point 1d). Please see four directed acyclic graphs (DAGs) below which we drew to show examples of potential mechanisms linking inflammation to IR and schizophrenia, which we have tested in our study. These are now included in the supplementary data file (S10 Methods) and referred to in the methods (P9 line 6) and discussion (P19 line 15; P19 line 20; P21 line 8).

In Panel A, inflammation is a common cause for IR and schizophrenia, which are not causally related to each other. In Panel B, IR mediates a causal relationship between inflammation and schizophrenia. In Panels C & D, similar mechanisms are considered with a reversed direction of association (i.e. schizophrenia is the causal agent).

Inflammation

Insulin Resistance

Schizophrenia

Inflammation

Insulin Resistance

Schizophrenia

Inflammation

Insulin Resistance

Schizophrenia

Inflammation

Insulin Resistance

Schizophrenia

A

B

C

D

In our study, we did not find evidence in bidirectional MR analysis that would support a causal effect of schizophrenia on IR, and so Panels C & D are not supported by our findings (as we describe on P19 line 15).

Additionally, we found only weak evidence which didn’t survive correction for multiple testing for a causal effect of HDL and triglycerides (an insulin resistance phenotype) with schizophrenia. Therefore, Panel B is likely not supported by our findings.

However, we found evidence in support of a causal relationship of inflammatory-related genetic variants for insulin resistance with schizophrenia, and the evidence for these associations attenuated fully to the null when we adjusted for CRP in MVMR sensitivity analysis, confirming that these associations are likely underpinned by inflammation. These findings are consistent with Panel A, that inflammation may be a common cause for schizophrenia and insulin resistance. This is outlined in the discussion (P19 line 16).

However, given the trend of evidence for IR and schizophrenia in the all-SNP analysis, we are unable to completely discount Panel B, and future GWAS with larger samples may help to clarify this, as we now specifically outline in the discussion (P21 line 7; P21 line 17).

**Reviewer:** c. In methods on page 8 the authors say that they repeat the analyses using only the inflammation-related traits in order to understand whether inflammation is a related mechanism. I am concerned that this, however, would simply capture the effect of inflammation on schizophrenia (more on this in point 3c below).  The rationale for and methods used in these analyses should be explained more clearly.

**Reply:** In our analysis of the inflammatory-related genetic variants for cardiometabolic traits, the genetic variants included were associated at genome-wide level with *both* the cardiometabolic trait and inflammation. Therefore, in that analysis, the effect of inflammation *and* the cardiometabolic trait is captured. With reference to our DAGs presented above and now included in S10 Methods, as described above, our findings are most consistent with Panel A, suggesting that inflammation is a common cause for IR and schizophrenia.

**Reviewer:** d. I am not clear as to how the multivariable MR was conducted. It is my understanding, that in these analyses one should include all SNPs from both traits, using the shared SNPs as instrument in order to derive direct effect of each exposure. However, here this does not seem to have been done (or, if it has, it is not clear)? I would have expected to see the univariable coefficients for CRP and IR, as well as the mutually adjusted ones.

**Reply:** We are grateful for the opportunity to clarify. The reviewer correctly describes the method of MVMR, which we followed. We have updated our methods to clarify that we did include CRP as an exposure in MVMR analysis (P11 line 16; S19 Methods). We did not present univariable MR results for CRP since this was not a goal of the study, and we did not hold specific hypotheses for CRP. Rather, we used CRP as a general measure of systemic inflammation as clarified above. Univariable MR results for CRP have been published (Hartwig et al, 2017) and replicated (Lin et al, 2019) elsewhere, and we reference these studies in our manuscript. Our MVMR mutually-adjusted point estimates for CRP are consistent with previously published univariable MR estimates. We have updated S1-2 Results for clarity and have referenced the previous studies which have examined CRP and schizophrenia in univariable MR analysis in footnotes in S1-2 Results.

**Reviewer:** e.  I am not sure I follow the results presented in Figure 3. Here, the authors show the main analyses adjusted for CRP. These don't change much, which is to be expected as there is no overall association and CRP has a negative association with Schizophrenia (see:(Hartwig et al. 2017)). However, the authors also present the inflammation-related SNPs adjusted for CRP, which - on the other hand - show a reduction in association. I wonder if this is not to be expected though as CRP will be correlated with other inflammatory markers and CRP has a negative association with SCZ. I think the most interesting finding in figure 3 is the first two lines of each trait showing an overall lack of association.

**Reply**: The reviewer is correct that CRP is likely to be correlated with other inflammatory markers since it is a general downstream marker of inflammation, and this is why in MVMR we controlled for CRP. The aim for the MVMR analysis was to show that MR associations for inflammation-related genetic-variants for a particular trait (e.g., HDL) and schizophrenia completely attenuated after controlling for CRP, providing further evidence that these associations are indeed underpinned by inflammation, rather than any other mechanisms.

We are now clearer to describe that we used CRP because it is a generalized marker of inflammation (P11 line 13; P20 line 2), rather than because we held specific hypotheses for the role of CRP in particular (P20 line 15). We used CRP in MVMR sensitivity analysis to further check whether any associations of inflammation-related cardiometabolic variants with schizophrenia were indeed likely driven by inflammation, rather than any other mechanism(s). We have clarified this within the methods section (P11 line 9). That the associations attenuated after adjusting for CRP suggests that the associations of inflammation-related variants were indeed driven by inflammation.

**Reviewer:** 3. Interpretation of results: a. In the discussion, the authors say that their study shows some evidence of an association between triglycerides, HDL, and leptin and SCZ. However, there seems to be little to no evidence of an association with these markers based on the data presented in table 1 (particularly after correction for multiple testing).

**Reply:** The reviewer is correct that the results for MR analyses using all SNPs for triglycerides and HDL show little evidence for association with schizophrenia. We have clarified this point in the results (P12 line 4). We have made similar amendments for leptin, which was more consistent across MR methods but still did not survive correction for multiple testing (P12 line 8). In addition, we have updated our discussion to clarify the interpretation (P19 line 6; P19 line 20; P22 line 8) and have made suggestions that larger GWAS studies in future may be helpful to confirm our findings (P21 line 19; P22 line 15).

**Reviewer:** b. On page 19, lines 11-12, the authors repeat that they wanted to test whether inflammation was a mechanism linking IR to schizophrenia. However, it is not clear how this can be hypothesized in the absence of a main effect between IR and schizophrenia.

**Reply:** As discussed in detail in response to comment 2b, by “mechanism linking” we mean common mechanism/cause. We agree that use of the term “linking” could be confusing as this might indicate presence of mediation, which, we also agree, would be unwise to test for in the absence of a main effect of the exposure on the outcome. We have now revised this sentence accordingly (P19 line 6). We hope the DAGs included in the paper now help to clarify different scenarios regarding associations between inflammation, IR and schizophrenia. As described earlier, our results are most consistent with Panel A from the four DAGs, suggesting that inflammation could be a common cause for schizophrenia and IR.

As mentioned in response to the previous comment, the DAGs are now included in the supplementary data file (S10 Methods) and referred to in the methods (P9 line 6) and discussion (P19 line 15; P19 line 20; P21 line 8).

**Reviewer:** c.  On page 19, the authors say that their "results are consistent with the idea that IR may be associated with schizophrenia over and above the effect of confounding from socio-demographic and lifestyle factors and that inflammation might be a common mechanisms". I am not sure how this inference was made, as there is no evidence of an association between IR and SCZ in the analyses. Showing an association between inflammation-related SNPs and SCZ only seems to validate the hypothesis of an inflammation (likely IL6 or other cytokines) and SCZ association? 

**Reply:** We agree with the reviewer that our results are consistent with inflammation being a common causal mechanism for IR and schizophrenia; we have now revised the conclusion accordingly (P24 line 14).

**Reviewer:** Minor concerns: 4.  On page 7, the authors explain that they use different MR methods depending on number of SNPs available. It would be helpful if they could add next to each of them the exposure they refer to. For instance, when they say "when <2 SNPs available" which traits are these? From the tables in the following pages I can't find any trait that only has one SNP.

**Reply:** We thank the reviewer for highlighting this. Our *a priori* statistical analysis plan proposed the use of Wald Ratio as a method for MR analysis in cases where only 1 SNP was available for a trait. The reviewer is correct that all included traits had more than 1 SNP available for analysis, and so we have removed this sentence from the methods to avoid confusion.

**Reviewer:** 5.  Please note that the sentence on page 7 lines 11-12 is incorrect. In a logistic regression, it is the log odds of the outcomes that are modeled, not the exposure. Also, similar to my previous comment, it would be helpful if the authors provided, in brackets, which exposures they are referring to, as this is not clear. 

**Reply:** We agree with the reviewer that in a logistic regression, the log odds of the outcome are modelled, and this is what we intended to convey. The sentence which we think the reviewer is referring to relates to our description of what the estimates represent for binary exposures (in this case, only type 2 diabetes mellitus), since the binary exposure would also have been modelled on a log scale. We agree that it would be helpful for clarity to list the exposures we are referring to and we have made this adjustment in the text (P9 line 22; P9 line 25). We hope the revised sentence is now clearer “For binary exposures (T2DM), the estimates represent the OR for schizophrenia per unit increase in the log-odds of T2DM”.

**Reviewer #3**

**Reviewer:** This paper aims to understand causal relationships between inflammation, cardiometabolic risk factors (especially the insulin resistance phenotype) and schizophrenia, which can have potentially important clinical values. The way they select SNPs to run MR is interesting, my main concern is in how the authors can properly and more clearly interpret their results using different MR methods. Here are my comments in details:

**Reply:** We thank the reviewer for their time and efforts, and for the insightful comments which have helped us to improve the manuscript.

**Reviewer:** 1. From the MR analyses that have been done in the paper, do the authors suggest the following causal mechanism where inflammation causally affect both schizophrenia and an IR phenotype while the latter two are not causally related themselves? If so, this seems to be a very interesting hypothesis, and I would hope the authors to more clearly describe how the MR analyses done in the paper indicate/suggest it. If not, then I'm wondering why the authors chose to use only inflammatory-related cariometabolic SNPs in the second set of analyses. From the paragraph in page 8, seems that the authors want to do detect "vertical pleiotropy", but it is unclear to me what vertical pleiotropy and for what trait the authors want to detect, and why using the inflammatory-related SNPs can achieve their goal.

**Reply:** We thank the reviewer for allowing us to clarify our hypothesis, analysis and approach to interpretation. The reviewer is correct that our conclusion; that inflammation causally affects both schizophrenia and IR, but the latter two are not causally related to each other, is the conclusion that is best supported by our findings. We have now added DAGs showing four potential scenarios regarding the association between inflammation, IR and schizophrenia, which we examined in our analysis. These are now included in the supplementary data file (S10 Methods) and referred to in the methods (P9 line 6) and discussion (P19 line 15; P19 line 20; P21 line 8):

Inflammation

Insulin Resistance

Schizophrenia

Inflammation

Insulin Resistance

Schizophrenia

Inflammation

Insulin Resistance

Schizophrenia

Inflammation

Insulin Resistance

Schizophrenia

A

B

C

D

In Panel A, inflammation is a common cause for IR and schizophrenia, which are not causally related to each other. In Panel B, IR mediates a causal relationship between inflammation and schizophrenia. In Panels C & D, similar mechanisms are considered with a reversed direction of association (i.e. schizophrenia is the causal agent). To specifically address the reviewers point on vertical pleiotropy, vertical pleiotropy would be consistent with DAGs B or D.

In our study, we found no evidence in bidirectional MR analysis suggesting a causal relationship association between schizophrenia and IR, and so Panels C & D are not supported by our findings (P19 line 15).

Additionally, we found only weak evidence which didn’t survive correction for multiple testing for a causal relationship HDL and triglycerides (an insulin resistance phenotype) and schizophrenia. Therefore, Panel B is likely not supported by our findings (P19 line 21).

Finally, we found evidence in support of a causal relationship of inflammatory-related genetic variants for insulin resistance with schizophrenia, and the evidence for these associations attenuated fully to the null when we adjusted for CRP in MVMR sensitivity analysis. These findings are consistent with Panel A, that inflammation may be a common cause for schizophrenia and insulin resistance. This is now outlined in the discussion (P19 line 20).

However, given the trend of evidence for IR and schizophrenia in the all-SNP analysis, we are unable to completely discount Panel B, and future GWAS with larger samples may help to clarify this, as we now outline in the discussion (P21 line 7).

**Reviewer:** 2. The authors used the word "association" and "no evidence of association" to describe their results in page 10 and 12, which I think is inappropriate. MR methods are for detecting causal relationships between trait, not just association. If the MR p-values are not significant, it does not mean "no evidence of association".

**Reply:** We agree with the reviewer that MR methods are for detecting causal relationships between traits, not just associations. However, following advice from the Editor to reduce the use of causal language, we have now revised the results section, where we talk about presence or absence of associations between genetically-predicted levels of cardiometabolic traits and schizophrenia, rather than causal or no causal relationships (P12 line 4 onward). However, we do use causal language in our interpretation of the results in the abstract and discussion (see abstract conclusion, discussion P19 line 6 onward).

**Reviewer:** 3. I did not see an MR analysis using Schizophrenia as the exposure, is it possible that Schizophrenia is a cause for the cardiometabolic risk factors?

**Reply**: The bidirectional MR analysis was carried out to examine schizophrenia as an exposure (as well as inflammatory-related schizophrenia SNPs) (P16 line 28). We have now revised the manuscript title to highlight this aspect. We did not find statistically significant MR associations of schizophrenia (exposure) with any cardiometabolic trait (outcome) after correction for multiple testing.

**Reviewer:** 4. From Table 2, there are very few inflammatory-related SNPs used. Looking into Figure 1, some significant results (such as for HDL) seem to be due to only one SNP. Since there are so few SNPs, the MVMR method may not have enough power to detect any effect after adding CRP. I think the authors may want to state the limitation.

**Reply:** We agree that with larger and better-powered GWAS studies, more SNPs and better SNP resolution may be achieved, which may in future may allow analysis with a greater number of SNPs overall, and possibly more inflammatory-related SNPs. We have added this point to the limitations (P23 line 24).

Additionally, we performed MR-PRESSO as a sensitivity analysis, which is able to detect the presence of pleiotropic outliers and perform analysis after removing any identified potential outliers. See S11 Methods for a detailed description of the method. We found little evidence of pleiotropic outliers for the majority of inflammatory-related cardiometabolic traits. Specifically, there was no evidence of pleiotropic outliers for FI, HDL or triglycerides, which were the inflammatory-related traits which returned evidence of a causal relationship with schizophrenia. Only T2DM and LDL returned evidence of potential outliers, and following outlier-corrected IVW, there was little change in estimates for either T2DM or LDL. Please see S10 Results for the full MR-PRESSO results for the inflammatory-related cardiometabolic SNPs.

**Reviewer:** 5. Related to 1, I'm wondering if showing CRP can be a cause of both schizophrenia and an IR phenotype using MR may strengthen the evidence that inflammation is the common mechanism.

**Reply:** We agree with the reviewer that showing evidence for causal relationships of CRP and indeed other inflammatory markers with schizophrenia and IR would be consistent with the findings we present in our study. Such analysis should ideally include a broad range of inflammatory markers including CRP to obtain a better understanding of network-level immune changes that causally influence risks for schizophrenia and cardiometabolic traits. We feel such extensive analyses should form the basis of a separate paper. This work is already underway within our group.

**Reviewer:** 6. In Table 1, are these number of SNPs the numbers after LD clumping? I'm guessing yes while wondering why the "53" number of SNPs for fasting insulin, TG and HDL keep the same after clumping.

**Reply:** We thank the reviewer for the opportunity to clarify. We have updated Table 1 so that the number of SNPs presented in the table is reflective of the SNPs that remained after clumping. This is now distinct from S1 Methods, where the table reflects the total number of GWAS-significant SNPs from the original GWAS. We have added a footnote to Table 1 to clarify this point.
