# Supplementary material for: The potential shared role of inflammation in insulin resistance and schizophrenia: A bidirectional two-sample mendelian randomization study
Source: PLoS Med. 2021 Mar 12;18(3):e1003455. doi: 10.1371/journal.pmed.1003455 (PMC7954314; doi:10.1371/journal.pmed.1003455)
Supplement: Attachment — Submitted filename: Response to Reviewer & Editor Comments.docx [file pmed.1003455.s037.docx]

**The potential shared role of inflammation in insulin resistance and schizophrenia: A bi-directional two-sample Mendelian randomization study**

Perry B.I. *et al*

**Response to Reviewer/Editor Comments and Production Requirements**

We are grateful to the editors and reviewers for their time and efforts in re-appraising our revised manuscript. Below, we present a list of editor/reviewer comments and production requirements alongside our responses to address each remaining comment. We submit two versions of the new amended manuscript; one clean copy and a second annotated copy with our changes highlighted. The annotated copy also includes line numbers, which we refer to in the comments below. We hope the manuscript is now suitable for publication.

**Comments From The Editor**

**Editor:** 1.Title: Please revise the title to: “The potential shared role of inflammation in insulin resistance and schizophrenia: A bi-directional two-sample Mendelian randomization study”

**Reply:** We have made the requested change (P1 line 1)

**Editor:** 2.Data availability statement: Please remove the section describing data access from the body of the manuscript and update the Data Availability Statement in the manuscript submission system.  This currently reads “Please see Data Availability Statement for information on downloading and accessing all publicly available data used in the manuscript.”
We suggest revising the text slightly for clarification: “The summary data for the 53 insulin resistance SNPs are available in the supporting information of Lotta et al [11] (10.1038/ng.3714). Full summary statistics for all traits used in the primary analysis are freely and publicly available for download at consortia/group websites. Specifically; for FI, FPG, HbA1C and glucose tolerance summary data, see <https://www.magicinvestigators.org/downloads/>; For HDL, LDL and triglycerides summary data, see <http://csg.sph.umich.edu/willer/public/lipids2013/>; For BMI summary data, see<https://portals.broadinstitute.org/collaboration/giant/index.php/GIANT_consortium_data_file>
s; For T2DM, see <https://diagram-consortium.org/downloads.html>; For leptin summary data, see
<ftp://ftp.ebi.ac.uk/pub/databases/gwas/summary_statistics/KilpelainenTO_26833098_GCST0>
03368; For schizophrenia summary data, see <https://www.med.unc.edu/pgc/download-results/>. Summary GWAS data for CRP, which formed part of our post-hoc sensitivity analysis, are also publicly available from the primary GWAS study [35], and inquiries regarding use of CRP summary data can be sent to [s.ligthart@erasmusmc.nl](mailto:s.ligthart@erasmusmc.nl).”

**Reply:** We have removed the Data Availability Statement from the body of the manuscript text and have added the suggested revised version of the statement into the appropriate section of the manuscript submission system.

Editor: 3.Abstract: Background, final sentence: Please avoid causal language here, we suggest revising to: “We aimed to examine whether there is genetic evidence that insulin resistance and seven related cardiometabolic traits may be causally associated with schizophrenia, and whether evidence supports inflammation as a common mechanism for cardiometabolic disorders and schizophrenia.”

**Reply**: We have made the suggested change (P2 line 7).

**Editor:** 4.Abstract: Methods and findings: Please provide some broad demographic information on the individuals represented in the various studies- particularly the numbers of included individuals.

**Reply:** We have made now added GWAS sample size to the Methods and findings section of the abstract (P2 line 14 onwards).

**Editor:** 5.Abstract: Methods and Findings, top of page 3: Should “was associated with schizophrenia” be “were associated with schizophrenia”?

**Reply:** We feel the sentence is correct as reported in the manuscript, i.e. “we report that a genetically-predicted inflammation-related insulin resistance phenotype… was associated with schizophrenia”. However, we appreciate that the sentence structure may have been confusing. We have revised the sentence with the use of brackets to aid interpretability of the sentence (P3 line 3).

**Editor:** 6.Abstract: Methods and Findings: Please revise to “We conclude that while our study did not find evidence in support of a causal relationship between schizophrenia and insulin resistance...” Also, this sentence could be moved to the “Conclusions” as this seems like more of an interpretation of the results.

**Reply:** We have revised this section for clarity. We have added to the section of the abstract reporting the results that “We did not find evidence in support of a causal relationship between cardiometabolic traits and schizophrenia, or *vice versa”* (P3 line 2), and have amended the first sentence of the conclusions section (P3 line 19).

**Editor:** 7.Abstract: Conclusions: Please revise the first sentence to avoid causal language: “"Our findings support a role for inflammation as a common cause for...".  Please also revise the final sentence, “...understand how inflammation may contribute to risk of schizophrenia and cardiometabolic disorders.”

**Reply:** We have made the requested changes (P3 line 19; P3 line 23). However, in this section, we have also replaced ‘cardiometabolic disorders’ with ‘insulin resistance’ for specificity (P3 line 22; P3 line 24).

**Editor:** 8..Author summary: Why was this study done?: Please revise the second bullet point: “Insulin resistance, a precursor to diabetes, is sometimes detectable in young adults suffering…”

**Reply:** We have made the requested change (P4 line 6).

**Editor:** 9.Author summary: Why was this study done?: There is a  typo in the third bullet point: “...and so could be a common mechanism for schizophrenia…”

**Reply:** We thank the editor for noticing this and have made the correction (P4 line 11).

**Editor:** 10.Author summary: What did the researchers do and find: Please revise the third and fourth bullet points as follows:
--After correction for multiple testing, overall, there was no significant evidence in support of a causal relationship between cardiometabolic traits and schizophrenia risk. However, we found evidence that supports a causal relationship between schizophrenia risk and an inflammation-related insulin resistance phenotype (comprising of raised fasting insulin, raised triglycerides and decreased high-density lipoprotein).
--Evidence for associations between schizophrenia risk and inflammation-related insulin resistance phenotypes attenuated fully in multi-variable MR analysis after adjusting for CRP, suggesting that these associations may be underpinned by inflammation.

**Reply**: We have amended the third and fourth bullet point as requested (P5 line 4; P5 line 10), however we have made a few minor tweaks to the proposed text. We have added *vice versa* (P5 line 6) since no significant evidence was found overall in either direction of association between cardiometabolic traits and schizophrenia. Additionally, in both revised bullet points, we have described the associations with the inflammation-related insulin resistance phenotype (exposure) preceding schizophrenia (outcome), since that was the direction of association we refer to in the bullet points. We hope this is acceptable.

**Editor:** 11..Author summary: What do these findings mean?: Please combine and revise the first and second bullet points as follows:
--These results suggest that cardiometabolic traits are unlikely to have a causal role in the pathogenesis of schizophrenia, and vice versa.  However, our results suggest that inflammation is related to the risk of both schizophrenia and cardiometabolic disorders, which may at least partly explain why they commonly occur in clinical practice.
Please revise the last two bullet points as follows: 
--Treating or preventing inflammation may be a putative therapeutic option for prevention and/or treatment of both schizophrenia and comorbid cardiometabolic disorders.
--In the future, more research is needed to understand the biological mechanisms underpinning how inflammation may increase the risk of schizophrenia and cardiometabolic disorders.

**Reply**: We have made the requested amendments (P5 line 14), however we have made tweaks for specificity, by replacing ‘cardiometabolic disorders’ with ‘insulin resistance’ (P5 line 16; P5 line 19; P5 line 21). We hope this is acceptable.

**Editor:** 12. Introduction Page 6: Background should be renamed “Introduction”

**Reply**: We have made the requested change (P6 line 1).

**Editor:** 13. Introduction: Page 7: Please revise to “...we have conducted a study to examine evidence in support of causality and shared mechanisms by testing the associations between IR and related cardiometabolic traits with schizophrenia.”

**Reply:** In light of comments from Reviewer 2, we have revised this section of the manuscript. However, we have followed the same style of language as suggested by the editor: “Therefore, we have conducted a study to examine evidence in support of four scenarios regarding the potential relationships between inflammation, IR and schizophrenia” (P7 line 15).

**Editor:** 14. Methods: Page 9: “Our analysis plan was prospectively conceived by the authors in 2019”  Please include the analysis plan as a supporting information file.  If a documented plan is not available to include, please mention that the all described analyses were planned, or mention which were planned and when/why any data-driven changes to analyses took place, including changes made in response to peer review comments.

**Reply:** We did not create a documented analysis plan, however we have made the suggested additions to the relevant section, now reporting “All described analyses were planned except the MVMR analysis including CRP (see below), which was conceived in light of the findings from the primary analysis, to test whether inflammation may be responsible for the results.” (P9 line 8). In the relevant methods section reporting the MVMR analysis including CRP, we also describe the analysis as “a *post-hoc* sensitivity analysis” (P11 line 12).

**Editor:** 15. Results: Page 12, first and second sentence: Please revise to “We did not find significant associations between genetically-predicted levels of triglycerides and HDL with schizophrenia…” and “We also observed an apparent association between genetically-predicted leptin levels and schizophrenia, but this was inconsistent across MR methods and the evidence did not survive correction for multiple testing”

**Reply:** We have made the requested change with a minor tweak. We now report “We did not find statistically significant evidence for associations between genetically-predicted levels of triglycerides and HDL with schizophrenia.” (P12 line 8). We hope this is acceptable.

**Editor:** 16. Discussion: First paragraph, first sentence: Please revise to reduce causal language: “...to first examine whether there are associations that support a causal relationship between IR and related cardiometabolic traits and schizophrenia, and second, to examine whether there is evidence supporting that inflammation may be a shared causal mechanism for IR and schizophrenia.”

**Reply:** We have made the requested amendment (P19 line 4).

**Editor:** 17. Discussion: First paragraph, second sentence: Please revise to: “We report that the evidence in support of a causal association between genetically-predicted levels of triglycerides, HDL and leptin and schizophrenia was weak, in that the evidence of these associations did not survive correction for multiple testing, estimates were inconsistent across MR methods, and may have been affected by horizontal pleiotropy.”

**Reply:** We have made the requested amendment (P19 line 7).

**Editor:** 18. Discussion: Page 19-20: Please revise to “In those analyses, we found strong and consistent evidence supporting that inflammation-related IR may have a causal relationship with schizophrenia.”

**Reply:** We have made the requested amendment (19 line 23).

**Editor:** 19. Discussion: Page 20: Please revise, as this is perhaps a likely interpretation but it cannot be said to be confirmed to be true: “This result suggests that the observed associations for the inflammatory-related variants are at least in part explained by inflammation.”

**Reply:** We have made the requested amendment (P20 line 3).

**Editor:** 20. Discussion: Page 21: Please revise to: “The implications of our findings with regard to shared causal mechanisms should not distract clinicians from focusing on the assessment and management of malleable lifestyle factors related to cardiometabolic disorders in people with schizophrenia.”

**Reply:** We have made the requested amendment (P21 line 21).

**Editor:** 21. Discussion: Page 23-24: Please edit this sentence to read: “In the future, larger and better-powered GWAS…”

**Reply:** We have made the requested amendment (P23 line 24).

**Editor:** 22. Reference list: Please double check the formatting for all references, but particularly Ref 28, 36, and please see our website for reference guidelines <https://journals.plos.org/plosmedicine/s/submission-guidelines#loc-references>

**Reply:** We have amended references 28 and 36 as per journal guidelines and have ensured the EndNote PLoS plugin we use coincides with journal guidelines.

**Editor:** 23. Checklist: Thank you for including the STROBE-MR checklist and we agree this is an appropriate checklist for your study.  However, it seems like the STROBE-MR is still under development/in the preprint stage (<https://peerj.com/preprints/27857v1/>) and for that reason please also include the STROBE checklist.

**Reply:** We have added an S2 Checklist – STROBE statement, referenced in the text (P10 line 15).

**Editor:** 24. Supporting information file/results:  It would be helpful to separate the supporting information items, particularly the results tables, into separate files.

**Reply:** We have now submitted supporting information items as individual files.

**Editor:** 25. S13 Results: Please spell out the abbreviation “NOME” in the title or legend.

**Reply:** We have made the requested amendment (S13 Results).

**Editor:** 26. S1 Figure: Please include a list of the beta coefficients with 95% CIs and p values unless they are already reported in another table.

**Reply**: Full numerical results for S1 Figure are presented in S3 and S4 Results. We have also added further information to the footnote of S1 Figure to make this clear: “See S3 and S4 Results for full numerical results for the IVW analysis presented in this figure alongside other MR analysis methods”.

**Comments from Reviewers**

**Reviewer #2**

**Reviewer:** The authors have addressed most comments. I still have a couple of suggestions concerning the causal hypotheses in the introduction, as I don't think these come across very clearly at the moment.

**Reply:** We thank the reviewer for their time and efforts in re-appraising our manuscript.

**Reviewer:** On page 7 the authors say "Inflammation could be a shared mechanism for cardiometabolic disorders and schizophrenia." Usually the term mechanism is used to describe causal pathways, whereas here it seems to be used to refer to the concept of confounding (i.e. inflammation causing both IR and SCZ). However, the authors then proceed to explain that they are testing whether IR is causally associated with SCZ (page 6) and whether "inflammation could be a shared mechanism linking IR and schizophrenia". If Inflammation is a common cause (i.e.: they hypothesise there is no effect of IR on SCZ after accounting for it), then IR cannot be said to be 'causally' associated with SCZ. As a result, at the moment it is still quite unclear what the causal hypotheses of these analyses are. Perhaps the authors should more clearly mention that they are testing 4 competing hypotheses and refer to the figure included in the revision already in the introduction.

**Reply:** As the reviewer correctly points out, we aimed to test four potential scenarios regarding the associations between inflammation, insulin resistance (IR) and schizophrenia, as represented by the panels in the DAGs. Different causal pathways are implicated in each DAG. For example, in Panel A, a causal pathway exists between inflammation and IR, and between inflammation and schizophrenia. In Panel B, a causal pathway exists between inflammation and IR, and between IR and schizophrenia. Panels C and D are similar, but with a reversed direction of association between IR and schizophrenia (i.e., schizophrenia as the causal agent for IR). Since we are testing different combinations of causal pathways, we have revised the use of ‘common mechanism’ where referenced by the reviewer. The sentence now reads “Inflammation could be pathophysiologically related to cardiometabolic disorders and schizophrenia” (P7 line 6). Later in the same paragraph we have now made a similar revision and additional clarification “However, to our knowledge, no MR studies have examined whether inflammation could be pathophysiologically related to IR and schizophrenia, for example via mediating or common-causal mechanisms” (P7 line 12). In addition, we agree with the reviewer that it would aid clarity to present the hypotheses (and associated DAGs) in the introduction rather than the methods section. We have amended our introduction in light of this (P7 line 15).

**Reviewer:** Related to the DAGs, I believe that in Panel A and Panel C there should also be an arrow between IR and SCZ (panel A) and IR and inflammation (panel C), as these describe confounding (e.g.: the authors test the association between IR and SCZ, but then test the role of inflammation as confounder)?  There should be one in the other two as well, as I think it cannot be hypothesised prior to testing that indirect effect=total effect (i.e. there is no direct effect, once including the mediator).

**Reply**: We thank the reviewer for pointing this out. We have added lines between the exposure and outcome (X and Y) in each DAG panel (S1 Methods).

**Reviewer #3**

**Reviewer:** The authors have addressed all my earlier comments.

**Reply:** We thank the reviewer for their time and efforts in re-appraising our manuscript.

**Production Requirements**

**Production:** FIGURES and GRAPHICS: 1. Please ensure that all main figures are referenced in the manuscript as Fig 1, Fig 2, etc. (including capitalization), rather than Figure 1, Figure 2, etc. Please note, however, that the file names themselves must not include the space, i.e. Fig1.tif, Fig2.tif. Please ensure that all main figure files are clearly labeled as such in the File Inventory.

**Reply:** We have renamed the figure files as per requirements and have amended figure referencing within the manuscript (P14 line 7; P15 line 10; P16 line 1; P16 line 14; P16 line 16).

Production: METADATA: 1. Please accurately complete the 'Competing Interests' section on our submission form, including any COIs declared by your co-authors. If you have no competing interests to declare, please add "The authors have declared that no competing interests exist". Otherwise please declare all competing interests beginning with the statement "I have read the journal's policy and the authors of this manuscript have the following competing interests:"

**Reply:** We have made the requested change.

**Production:** 2. Your co-author, Dr. Jones, has declared the following competing interest in relation to this manuscript. Please include it in the main competing interest statement in our system, sticking as closely as possible to Dr. Jones's wording, using only their initials. 
"I have received honoraria for providing scientific advice to Jansen, Ricordati and Lundbeck"

**Reply:** We have made the addition.

**Production**: 3. Please ensure only the authors' initials are included in the competing interest statement, not their full names or titles.

**Reply**: We confirm this is the case.

**Production:** 4. Please provide a detailed Financial Disclosure statement. This is published with the article, therefore should be completed in full sentences and contain the exact wording you wish to be published. - Please clarify all sources of funding (financial or material support) for your study. List the grants (with grant number) or organizations (with url) that supported your study, including funding received from your institution. 
- State the initials, alongside each funding source, of each author to receive each grant.
- State what role the funders took in the study. If the funders had no role in your study, please state: “The funders had no role in study design, data collection and analysis, decision to publish, or preparation of the manuscript.”
- If any authors received a salary from any of your funders, please state which authors and which funders.
If you did not receive any funding for this study, please state: “The authors received no specific funding for this work.”

**Reply:** We have made the requested change.

**Production:** 5. In accordance with PLOS' data policy, please ensure that your Data Availability Statement in the submission form clearly identifies how readers can access your data. Note that it will be typeset as it is written so please ensure it is in complete sentences and appears as you would like it to in the published version.
For details on our data policy, please see <http://journals.plos.org/plosmedicine/s/data-availability>

**Reply**: We have revised our Data Availability Statement as per editorial request. The Data Availability Statement clearly identifies how readers can access the data, and is presented in complete sentences.

**Production:** ARTICLE FILE: 1. Please indicate the corresponding author on the title page with an asterisk (*) and include the corresponding author's email address. Only an asterisk and the email address itself are required.

**Reply:** We have made the requested change.

**Production:** 2. Please remove author degree titles from the byline of your manuscript file.

**Reply:** We have made the requested change.

**Production**: 3. Please ensure that all author names are spelled correctly in the manuscript file and the submission form, including the use of a middle name/initials, and any special characters (accents, umlauts, etc.). Please place first/given names before last/family names. We ask that you ensure they match verbatim. Please be aware that correct spelling and formatting of author names in the article file is vital for accurate indexing in PubMed. The following author names do not match verbatim across the article file and the submission form: Benjamin I. Perry; Felix Day

**Reply**: We have made the requested changes.

**Production:** 4. Please ensure that your Ethics Statement is available in the Methods section of your manuscript in its entirety, under the subheading "Ethics Statement".

**Reply:** We have made the requested change (P9 line 1).

**Production:** 5. We have noted that you have the following present in your manuscript: Competing Interests statement/ Data Availability Statement. We will remove these section from your manuscript file as this information is automatically pulled from the submission form. Please make sure that the information in the submission form is correct.

**Reply:** We have removed these sections from the manuscript file.

**Production:** SUPPORTING INFORMATION: 1. Please upload your Supporting Information items individually, rather than in a single file, as you refer to them individually in the text. If you would like to retain the single file please rename internal items (e.g. tables) as A, B, C etc. Please also provide a legend for each under the heading Supporting Information. Supporting Information files should be less than 10 MB each.  Please edit any citation of internal items within your Supporting Information files to using letters rather than numbers, e.g. “Table A in S1 Text”. Please ensure these changes are made throughout the manuscript and within the Supporting Information files themselves.

**Reply:** We have now submitted supporting information items individually.

**Production:** 2. Please ensure that all Supporting Information files use the following format verbatim throughout the manuscript (including legends and all in-text citations): S1 Fig, S1 Table, S1 Text, S1 Data, etc. Each file type should start at 1 and increase numerically. Please note, however, that the file names themselves must use an underscore rather than a space, i.e. S1_Fig.tif, S1_Table.xlsx, S1_Text.doc, S1_Data.xlsx, S1_PRISMA_Checklist.docx, etc.

**Reply:** We have made the requested changes.

**Production:** 3. Please ensure that each supporting information file has a legend at the end of the manuscript file, after the Reference list.

**Reply:** We have moved the Supporting Information Captions section to the end of the manuscript file (P29 line 12).

**Production:** 4. Please note that your submission cannot contain any references to generic "supporting information" files and instead must refer to specific files using our naming convention (S1 Fig, S1 Table, S1 Text, S1 Data, etc.)

**Reply:** We confirm that we do not make reference to generic supporting information files.
